# Engineering *Aspergillus oryzae* for the Heterologous Expression of a Bacterial Modular Polyketide Synthase

**DOI:** 10.3390/jof7121085

**Published:** 2021-12-17

**Authors:** Jin Feng, Maurice Hauser, Russell J. Cox, Elizabeth Skellam

**Affiliations:** 1 Institute for Organic Chemistry and Biomolekular Wirkstoff Zentrum, Leibniz University Hannover, Schneiderberg 38, 30167 Hannover, Germany; jin.feng@oci.uni-hannover.de (J.F.); maurice.hauser@oci.uni-hannover.de (M.H.); 2 Department of Chemistry, BioDiscovery Institute, University of North Texas, 1155 Union Circle, Denton, TX 76201, USA

**Keywords:** propionyl-CoA metabolism, modular polyketide synthase, propionyl-CoA carboxylase, heterologous expression

## Abstract

Microbial natural products have had phenomenal success in drug discovery and development yet form distinct classes based on the origin of their native producer. Methods that enable metabolic engineers to combine the most useful features of the different classes of natural products may lead to molecules with enhanced biological activities. In this study, we modified the metabolism of the fungus *Aspergillus oryzae* to enable the synthesis of triketide lactone (TKL), the product of the modular polyketide synthase DEBS1-TE engineered from bacteria. We established (2S)-methylmalonyl-CoA biosynthesis via introducing a propionyl-CoA carboxylase complex (PCC); reassembled the 11.2 kb DEBS1-TE coding region from synthetic codon-optimized gene fragments using yeast recombination; introduced bacterial phosphopantetheinyltransferase SePptII; investigated propionyl-CoA synthesis and degradation pathways; and developed improved delivery of exogenous propionate. Depending on the conditions used titers of TKL ranged from <0.01–7.4 mg/L. In conclusion, we have demonstrated that *A. oryzae* can be used as an alternative host for the synthesis of polyketides from bacteria, even those that require toxic or non-native substrates. Our metabolically engineered *A. oryzae* may offer advantages over current heterologous platforms for producing valuable and complex natural products.

## 1. Introduction

**Figure 1:**
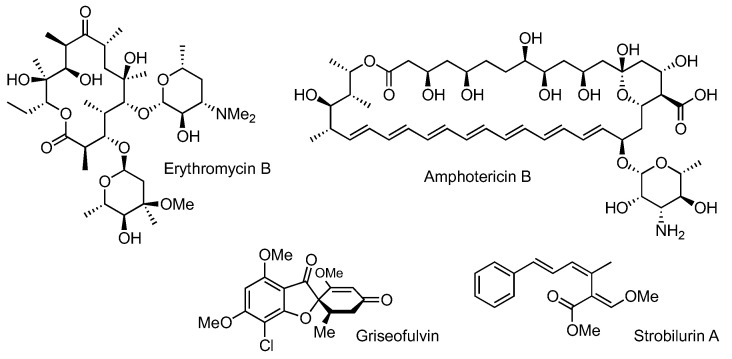
Examples of bioactive polyketide natural products from bacteria and fungi.

Recent estimates suggest that over 25% of drugs approved between 1981 and 2014 are natural products or directly derived from natural products [1]. The bacterial polyketide class of compounds constitutes a significant percentage of natural products used in clinical or agricultural settings. This class of compounds includes the antibiotic erythromycin B and the antifungal compound amphotericin B, among many others (Figure 1). Fungi are also prolific producers of valuable antibiotics such as the polyketides griseofulvin and strobilurin A. Industrial production of these compounds often relies on large-scale fermentation and semi-synthesis as the most efficient routes [2]. Thus, the development of new biosynthetic engineering platforms that can rationally manipulate and improve the production of these compounds is an important current goal.

**Scheme 1:**
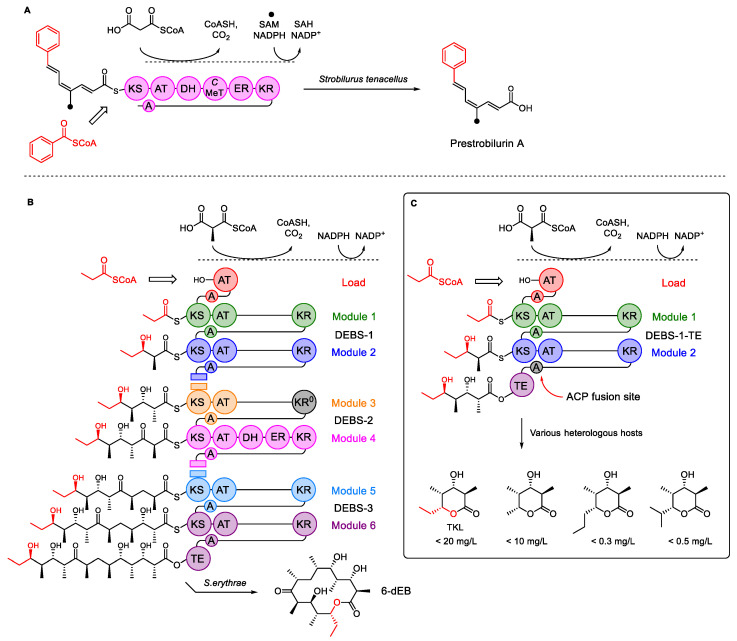
Type I PKS. (**A**) The iterative PKS involved in the biosynthesis of prestrobilurin A; (**B**) 6-module DEBS system that synthesizes 6-dEB; (**C**) engineered DEBS1-TE that synthesizes triketide lactones (TKL). Abbreviations: KS, ketosynthase; AT, acyl transferase; A, acyl carrier protein (ACP); DH, dehydratase; *C*-MeT, *C*-methyl transferase; ER, enoyl reductase; KR, keto-reductase; KR^0^-inactive KR domain; TE, thiolesterase. Diagrams inspired by Keatinge-Clay [3].

Polyketides are produced in two phases: a polyketide synthase (PKS) first assembles a carbon skeleton that is later modified by tailoring enzymes that can introduce a high diversity of chemical functionalization. Type I PKS consist of large multifunctional proteins with individual functional domains that are covalently linked. Type I PKS are further sub-divided into iterative PKS (iPKS) and modular PKS (mPKS, Scheme 1). iPKS consists of a single module (Scheme 1A), where each catalytic domain typically accepts, extends, and processes different substrates during each cycle of chain extension. iPKS use the same set of functional domains repeatedly until chain extension is complete.

In contrast, mPKS consists of multiple modules. Each module typically accepts, extends, and processes a single substrate, and passes it to the next module for further processing. Type I iPKS are typical of fungi but are also known in bacteria, while type I mPKS are almost exclusively limited to bacteria.

Heterologous expression has emerged as a highly successful strategy for the production and engineering of microbial natural products in both bacteria and fungi [4]. Heterologous expression systems usually rely on host strains that are phylogenetically close to the producing organism to increase the likelihood that native transcriptional elements function properly, promoters remain functional, translation is efficient, the resulting proteins fold correctly, and codon usage is relatively conserved. In addition, post-translational modification processes, such as phosphopantetheinylation of acyl carrier proteins (ACP), must remain effective. Phylogenetically diverse organisms are also used as heterologous expression hosts for complex polyketides. Such organisms include *Escherichia coli* [5] and *Saccharomyces cerevisiae* [6,7], however, extensive host-engineering is often required, and titers can be low [8].

*Aspergillus oryzae* is a filamentous fungus commonly used in the fermentation industry for the production of sake, miso, and soy sauce from rice [9]. It has a generally regarded as safe (GRAS) status and is easily cultured in the lab, amenable to genetic modification using a variety of techniques, and is known for its high protein production. Recently, *A. oryzae* has proven itself to be an extremely capable host for heterologous expression of complete and partial fungal biosynthetic gene clusters (BGC), and for their systematic engineering [10,11,12,13,14,15,16,17]. *A. oryzae* does not produce significant amounts of its own secondary metabolites, meaning it is a clean host allowing facile detection and purification of heterologously produced compounds. Moreover, there are a number of examples where heterologous expression of a specific pathway in *A. oryzae* has exceeded the titer of the natural product reported in the native producer [18,19]. Advantages of fungi over other heterologous hosts are: the use of monocistronic operons, meaning separate genes can be individually controlled; numerous cellular compartments, where selective reactions may be contained to overcome toxicity; and tailoring enzymes that catalyze diverse and unique chemical modifications not accessible to other organisms [20]. However, *A. oryzae* has not yet been explored as a host for the production of bacterial polyketides. In particular, it is notable that reports of the presence of modular PKS in fungi are extremely rare [21]. However, successful expression of modular PKS in fungi could open up significant opportunities for the production of hybrid bacterial-fungal compounds unknown in nature, with potentially unique properties. In addition, fungi are capable of numerous oxidative tailoring modifications unknown in bacteria, offering further possibilities for the generation of novel compounds [22].

The biosynthesis of 6-deoxyerythronolide B (6-dEB) in *Saccharopolyspora* *erythraea* has long been used as a model mPKS system. Here, three large multimodular proteins (DEBS1, DEBS2, and DEBS3, Scheme 1B) work together to make the hexaketide macrolide 6-deoxyerythronolide B (6-dEB). DEBS1-TE is a well-studied simplified mPKS derived by fusing the C-terminal thiolesterase (TE) release domain of DEBS3 to the C-terminus of DEBS1 [23]. DEBS1-TE thus consists of a loading module and two extending modules that synthesize triketide lactone (TKL) from propionyl CoA and 2*S*-methylmalonyl CoA (Scheme 1C). The observed titers vary (0.5–20 mg·L^−1^) depending on the host used (Scheme 1) [24,25,26,27]. The acyltransferase (AT) domain within the DEBS1-TE loading module usually selects and activates propionyl-CoA, although other small acyl CoAs can be used if propionate is lacking.

A number of factors are likely to impede the use of fungal hosts for the successful expression of mPKS. For example, high concentrations of propionyl CoA are toxic to fungi through inhibition of crucial metabolic pathways [28,29] and 2*S*-methylmalonyl-CoA, required by mPKS extender modules, is not known to be synthesized by fungi. Additionally, actinobacterial genes are typically high GC% and it is unknown whether transcription and translation would be effective in *A. oryzae*. Furthermore, DEBS1-TE requires post-translational modification of the ACP domains by a phosphopantetheinyl-transferase enzyme (PPTase), and it is unknown whether the native *A. oryzae* PPTase is capable of post-translationally modifying the ACP domains of mPKS.

In this study, we modified the metabolism of *A. oryzae* to enable the synthesis of TKL by the DEBS1-TE modular PKS. We established 2*S*-methylmalonyl-CoA biosynthesis *via* the introduction of a bacterial propionyl-CoA carboxylase complex (PCC); reassembled the 11.2 kb DEBS1-TE-coding region from synthetic codon-optimized gene fragments using rapid yeast recombination; introduced the bacterial phosphopantetheinyltransferase (PPTase) SePptII; investigated the propionyl-CoA synthesis and degradation pathways; and developed improved delivery of exogenous propionate. Overall, we demonstrated that *A. oryzae* can be used as an effective alternative host for the synthesis of bacterial polyketides that require toxic or non-native substrates, requiring minimal metabolic modification. *A. oryzae* may thus offer future advantages over other heterologous platforms for producing valuable and complex bacterial natural products.

## 2. Materials and Methods

See Supplementary Material for tables of media, strains, plasmids, and oligonucleotides.

### 2.1. Strains and Culture Conditions

One Shot^TM^ Top10 chemically competent *Escherichia coli*, One Shot^TM^ *ccdB* Survival^TM^ 2 T1^R^-competent *E*. *coli*, and One Shot™ OmniMAX™ 2 T1^R^ *E*. *coli* were used as hosts for the construction of general plasmids and grown in LB medium with 50 mg·mL^−1^ of the antibiotic carbenicillin at an incubation temperature of 37 °C. *Saccharomyces cerevisiae* strain CEN.PK was used to prepare competent yeast cells in YPAD medium and then utilized as the host for the expression plasmid assembly by homologous recombination in SM-URA medium at 28 °C. *Aspergillus oryzae* NSAR1 was used as the heterologous host for fungal transformation and metabolite production in DPY medium at 28 °C.

### 2.2. Propionyl-CoA Toxicity Assessment to A. oryzae NSAR1

Methionine, isoleucine, arginine, and sodium propionate were individually dissolved in deionized water and sterilized. The prepared solutions were then mixed into DPY agar media at increasing concentrations of 0, 10 mM, 25 mM, 50 mM, and 100 mM, respectively. The same amount of *A. oryzae* spore suspension was inoculated on the face of each DPY plate bearing the supplementary compound. The culture plates were grown in the incubator at 28 °C. After 2, 4, and 7 days, the growth state of each sample was recorded. 

### 2.3. PCR-Based Gene Identification

Fungal genomic DNA for PCR was extracted from mycelia of *A. oryzae* NSAR1 grown in DPY medium by using the GenElute plant genomic DNA kit (Sigma-Aldrich, Darmstadt, Germany). Using genomic DNA as the PCR template, a pair of specific 5′ and 3′ primers for gene identification were designed. PCR product purification was carried out by using the Wizard SV gel and PCR clean-up system (Promega, Madison, WI, USA). The PCR product was further identified by gene sequencing.

Total RNA was isolated from fresh 2-day-old cultured mycelia of *A. oryzae* NSAR1 grown in DPY medium by using the Quick-RNA™ Fungal/Bacterial Miniprep Kit according to the manufacturer′s protocol (Zymo Research, Irvine, CA, USA). Single-stranded cDNA was prepared from total RNA by using the High-Capacity RNA-to-cDNA™ Kit (ThermoFisher, Waltham, MA, USA). Using the cDNA as a template, the PCR reaction with specific 5′ and 3′ primers for gene identification was carried out. For the gene identification under the condition of adding propionate, 50 mM of sodium propionate was supplemented into the *A. oryzae* culture one day before total RNA isolation. 

### 2.4. Construction of pTYGS·arg·pccABE

The vector pTYGS·*arg* was fully digested using *Asc*I. All codon-optimized *pcc* gene fragments (http://genomes.urv.es/OPTIMIZER/, accessed on: 1 January 2020) flanked with overlaps (by *ca* 30 bp) were synthesized commercially. The plasmids bearing *pccABE* gene fragments were prepared by using double restriction enzymatic digestion (*pccA*-*EcoR*I/*BamH*I, *pccB*-*Xba*I/*BamH*I, *pccE*-*BamH*I/*Xba*I) for the plasmid reassembly in yeast. The competent *Saccharomyces cerevisiae* CEN.PK cells were removed from −80 °C storage and placed on ice to thaw. The following components were added to the yeast pellet in order: 240 μL PEG solution (50% (*w*/*v*) polyethylene glycol 3350); 36 μL LiAc (1 M); 50 μL denatured salmon testis DNA (2 mg·mL^−1^ in TE buffer) and 34 μL DNA fragments containing the linearized pTYGS·*arg* and three desired inserts in equimolar concentration (the uncut plasmid was used as the positive control and the linearized plasmid was used as the negative control). The mixture was resuspended and incubated for 50 min at 42 °C. Cells were pelleted by centrifugation at 11,000× *g* for 15 s and the supernatant was removed. The pellet was resuspended in 500 μL deionized H_2_O, and 100 μL suspension was spread on selective SM-URA plates, which were incubated for 3 days at 28 °C. 

Yeast colonies were gathered. Plasmids were extracted from yeast colonies by using the Zymoprep^TM^ Yeast Plasmid Miniprep II kit (Zymo Research, Freiburg Germany). Subsequently, using standard heat-shock protocols, the extracted plasmid mixture was introduced into *E*. *coli ccdB* survival 2 T1^R^ with the antibiotic carbenicillin (50 mg·mL^−1^). Large amounts of *E*. *coli* colonies were generated, and then five colonies were picked up and identified by the colony PCR using specific 5′ and 3′ test primers. Plasmids were then extracted from positive colonies by using the Nucleospin^®^ Extract Kit (Machery-Nagel, Düren, Germany) and identified using the double restriction enzymatic digestion (*Nhe*I+*Swa*I). The construct was checked by gene sequencing for correct construction. 

### 2.5. Construction of pTYGS·arg·debs1te·pccABE

DEBS1-TE, as a non-native gene, was designed in silico using data from its original construction by Leadlay and coworkers [23]. The whole DEBS1-TE sequence (11.2 kb) was codon-optimized (http://genomes.urv.es/OPTIMIZER/, accessed on: 1 January 2020). Meanwhile, the original start codon GTG was replaced with ATG. For synthetic convenience, the entire DEBS1-TE sequence was designed as four fragments (~2.8 kb each). Each fragment was designed to overlap with adjacent fragments or the expression vector at both ends (by *ca* 30 bp) for the subsequent yeast recombination. Two different unique restriction enzymes were adhered to ends of each fragment (*debs1te*-*f1*-*Sca*I/*BamH*I; *debs1te*-*f2*-*Sma*I/*Hand*III; *debs1te*-*f3*-*EcoR*V/*Xba*I; *debs1te*-*f4*-*Dra*I/*EcoR*I). 

The four DEBS1-TE fragments were synthesized commercially in four separate pUC57 vectors. All desired DEBS1-TE fragments and the linearized pEYA vector (fully digested by *Not*I) were prepared by restriction-enzyme digestion and then introduced into competent yeast for homologous recombination as described above. After two days of incubation, yeast colonies were collected. Plasmids were extracted from yeast colonies and immediately introduced into *E*. *coli* Top10 competent cells with the antibiotic kanamycin (50 mg·mL^−1^). *E*. *coli* colonies were screened by PCR identification. In the resulting positive *E*. *coli* colonies, plasmids were extracted and then identified by restriction enzyme digestion. The empty vector pEYA was used as the control. The extracted plasmids were verified by the restriction enzyme digestion using *Sca*I. The construct pEYA-*debs1te* was finally confirmed by gene sequencing for no mutation.

The pEYA·*debs1te* construct was then used as the entry vector and pTYGS·*arg*·*pccABE* was used as the destination vector in a Gateway in vitro LR recombination reaction according to the manufacturer′s instructions of the Gateway™ LR Clonase™ II Enzyme Mix Kit (Invitrogen). Transformation of the recombination mix into *E. coli* One Shot™ OmniMAX™ 2 T1^R^ with the carbenicillin selection afforded the plasmid construction, which was then confirmed as pTYGS·*arg*·*debs1te*·*pccABE* by colony PCR and restriction enzyme digestion as described above. Lastly, the construct was checked by gene sequencing for correct construction. 

### 2.6. PEG-Mediated Transformation of A. oryzae NSAR1

*A*. *oryzae* NSAR1 conidia from sporulating plates were inoculated into 50 mL GN medium and incubated overnight at 28 °C with 110 rpm shaking. Germinated conidia were collected and incubated in 10 mL of filter-sterilized lysing solution at room temperature with gentle mixing for 3 h. The protoplasts were released and filtered through a sterile Miracloth. The filtrate was centrifuged at 3000× *g* for 5 min to pellet the protoplasts which were resuspended in solution I. The plasmid pTYGS·*arg*·*debs1te*·*pccABE* (~1 μg) was added to 100 µL of the protoplast suspension and incubated on ice for 2 min. One milliliter of solution II was added and the transformation mixture was incubated at room temperature for 20 min. Five milliliters of molten CZD/S top medium with 0.8% agar was added to the transformation mixture and overlaid onto prepared CZD/S bottom medium plates with 1.50% agar. Plates were incubated at 28 °C for 3 days. For the introduction of co-expression plasmids pTYGS·*arg*·*debs1te*-*egfp*·*pccABE* and pTYGS·*met*·*sepptII*, the selection medium CZD/S without methionine was used in fungal transformation. After 3 days of incubation, the single transformant was transferred onto the new selection medium plate for 2 more rounds of screening. The pure transformants were inoculated onto the DPY plates for incubation at 28 °C for 5 days. Generated conidia were collected and transferred to the DPY liquid media for fermentation. 

### 2.7. TKL Fermentation, Extraction and Analysis

*A. oryzae* transformants were fermented in the liquid DPY media (100 mL in 500 mL Erlenmeyer flask) at 28 °C for 5 days. Sodium propionate was added to a final concentration of 50 mM, either in one batch, in five equal batches over 5 days, or continuously over 5 days by a syringe pump. Cultures were grown and harvested. Cultures (100 mL) were homogenized by an electric blender. Solids were removed by vacuum filtration. The filtrate was extracted two times with EtOAc (100 mL). The combined organic extract was dried (anhydrous MgSO_4_), filtered, and evaporated to dryness in vacuo and weighed. Methanol (1 mL) was added to dissolve the extract. The impurity was removed through spinning at 14,000× *g* for 5 min, and the sample was subjected to LCMS analysis. 

Analytical LCMS data for 20 μL samples were obtained using a Waters LCMS system consisting of a Waters 2767 autosampler, Waters 2545 pump system, a Phenomenex Kinetex column (2.6 μm, C_18_, 100 Å, 4.6 × 100 mm) equipped with a Phenomenex Security Guard precolumn (Luna, C_5_, 300 Å) at a flow rate of 1 mL·min^−1^. Detection was carried out by a diode array detector (Waters 2998) in the range 210 to 600 nm and an ELSD detector (Waters 2424) connected to a mass spectrometry, Waters SQD-2 mass detector, operating simultaneously in ES+ and ES- modes between 100 and 1000 *m/z*. The mobile phase was composed of HPLC-grade water mixed with 0.05% formic acid (solvent A) and HPLC-grade acetonitrile mixed with 0.045% formic acid (solvent B). A solvent gradient was run over 15 min starting at 10% B and ramping up to 90% B. In the case of the competition assay, a shallower gradient was applied ramping from 10 to 30% B in 15 min.

### 2.8. TKL Quantification 

The synthetic TKL was dissolved in methanol and diluted to different concentrations from 2.5 μg·mL^−1^ to 200 μg·mL^−1^. Each concentration was analyzed and the corresponding product peak on SIR (single ion recording) LCMS chromatogram was integrated. A calibration curve was made based on the concentrations and peak areas of the synthetic TKL. Then, a standard extraction workflow was determined. The transformant was inoculated in 100 mL DPY media. After 4 days of cultivation, 50 mM sodium propionate was added into the culture. The culture with propionate was incubated overnight. Next, the five-day culture was extracted with the same volume of ethyl acetate twice. The organic phase was gathered and evaporated to dryness. The concentrated extract was re-dissolved in 1.5 mL methanol. Subsequently, the extract suspension was diluted 10 times with methanol. The sample was centrifuged at 14,000× *g* for 5 min. Lastly, the supernatant was collected and analyzed by LCMS using the SIR detection method. The extract from 100 mL of the transformant culture was applied to the quantitative analysis based on the calibration curve. By calculation, the titer of the triketide lactone product TKL in *A. oryzae* was determined. 

### 2.9. Gene Knockout of Degradation Pathways

Specific primers for *degradation gene* knockout cassette construct were designed. Primers were used to individually amplify the 5′ and 3′ termini (750 bp each) of *degradation gene* from *A. oryzae* genomic DNA. The promoter *P_trpC_* and selection marker *argB* were also flanked by overlaps (30 bp) with *degradation gene* fragments by PCR. Then, all fragments were reassembled to the *Not*I-digested pEYA to make the *degradation gene* knockout cassette by yeast recombination. Subsequently, the bipartite gene knockout-based fungal transformation was carried out as previously published protocols. After transformant screening, single transformants were obtained followed by fluorescence imaging. Positive transformants were selected and subject to genomic DNA extraction. All genomic samples were identified by PCR using multiple specific primers. 

## 3. Results

### 3.1. Investigating Propionyl-CoA Metabolism in A. oryzae 

**Scheme 2:**
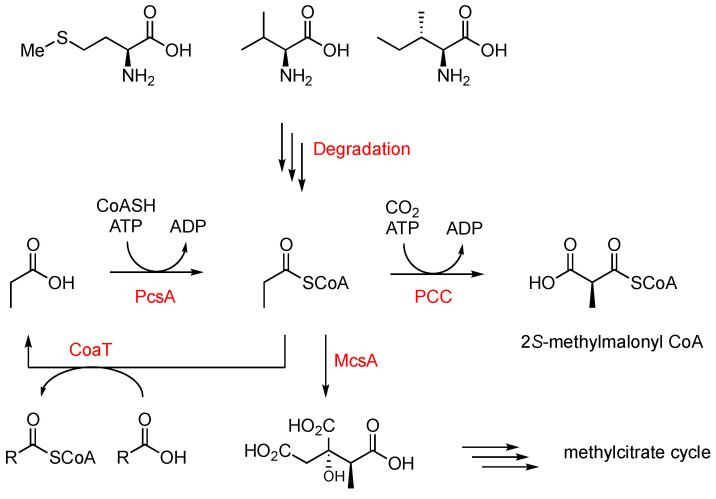
Propionate metabolism relevant to this work. Abbreviations: PcsA, propionyl-CoA synthetase; PCC, propionyl-CoA carboxylase, McsA, methylcitrate synthase; CoaT, acyl-CoA transferase.

Propionyl-CoA is synthesized in fungi *via* the degradation of odd-chain fatty acids, amino acids such as methionine, isoleucine, and valine, or by thiolesterification of exogenous propionic acid (Scheme 2). However, sodium propionate is also a useful antifungal agent because high levels of propionyl-CoA are toxic to many fungi due to the inhibition of pyruvate dehydrogenase and succinyl-CoA synthetase [28,29]. This inhibition affects glucose and acetyl-CoA metabolism, respectively. In order to test the toxic effects of elevated levels of propionyl-CoA, we grew *A. oryzae* on dextrin-peptone-yeast Extract (DPY) agar supplemented with varying concentrations (0–100 mM) of methionine, isoleucine, and sodium propionate. 

As a control, we also supplemented the medium with arginine, an amino acid not converted to propionyl-CoA. DPY is a rich medium often used with the pTYGS·*arg* heterologous expression system, developed specifically for *A. oryzae* NSAR1 [30].

**Figure 2:**
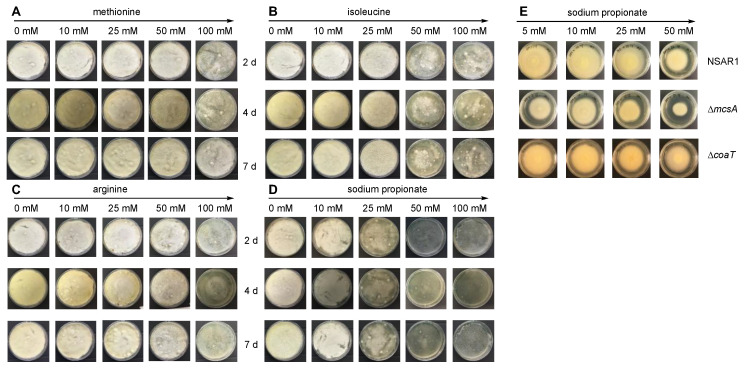
Susceptibility of *A. oryzae* NSAR1 to different amino acids and propionate at the indicated concentrations and times. (**A**) Methionine; (**B**), isoleucine; (**C**) arginine; (**D**) sodium propionate; (**E**) susceptibility of indicated mutants to indicated concentrations of sodium propionate after 5 days.

All supplements inhibited the growth of *A. oryzae* NSAR1 at 100 mM, evident from as early as two days (Figure 2A–D). The control, arginine, had the least effect on the growth of *A. oryzae* at any concentration, whereas methionine exhibited toxic effects at concentrations above 50 mM, isoleucine exhibited toxic effects at concentrations above 25 mM, and sodium propionate inhibited growth at 10 mM. At 25 mM, sodium propionate had a drastic effect on fungal growth and at concentrations above 50 mM sodium propionate completely abolished the ability of *A. oryzae* NSAR1 to grow on DPY. Since sodium propionate is the most direct precursor to propionyl-CoA and induces inhibitory effects at relatively low concentrations, we selected this to study propionyl-CoA metabolism in *A. oryzae* NSAR1.

In *Aspergillus nidulans*, propionyl-CoA is synthesized from propionate, coenzyme A, and ATP by propionyl-CoA synthetase (PcsA), encoded by *pcsA* (Scheme 2) [31]. *A. oryzae* NSAR1 is a quadruple auxotroph (*argB*−, *niaD*−, *sC*−, and *adeA*−) derived from parental strain *A. oryzae* RIB40, the genome of which is fully sequenced. *A. oryzae* ORF XP_001826479 was identified as the closest homolog of *pcsA* by direct BLAST query of the *A. oryzae* RIB40 genome. In *Aspergillus nidulans*, two routes are known for the degradation of propionyl CoA. First, methylcitrate synthase (McsA) condenses propionyl-CoA with oxaloacetate to yield 2*S*,3*S*-methylcitrate (Scheme 2) [32]. Second, a broadly selective CoA-transferase (CoaT) transfers CoASH from various acyl thiolesters to abundant carboxylic acids [33]. Both McsA and CoaT are compartmentalized in mitochondria [32,33]. *A. oryzae* RIB40 ORFs XP_001817006 and XP_001817633 were identified as *mcsA* and *coaT* homologs, respectively.

To determine if *A. oryzae pcsA* is specifically induced by propionate, we conducted RT-PCR from cDNA. Transcription of *pcsA* was detected when sodium propionate was added at 50 mM but not when omitted from solid growth medium (see Electronic Supplementary Information [ESI]). Likewise, *mcsA* could only be amplified from cDNA when propionate was added to the culture. In contrast, *coaT* could be amplified from cDNA without the addition of exogenous propionate (ESI). These results confirm that PcsA and McsA are induced by propionate, but CoaT is active regardless of the presence of propionate.

### 3.2. Introducing a Methylmalonyl-CoA Pathway into A. oryzae NSAR1

Confident that propionyl-CoA metabolism is active in *A. oryzae* NSAR1, and that the propionyl-CoA starter unit would be available for DEBS1-TE, we turned our attention to the production of 2*S*-methylmalonyl-CoA. We opted to add the well-understood bacterial propionyl-CoA carboxylase (PCC) pathway to *A. oryzae*. PCC consists of α, β, and ε subunits: the ε-subunit interacts with the β-subunit to dramatically increase the specificity of the enzyme complex [34]. A recent study of various PCC systems in *E. coli* indicated that PCC from *Streptomyces coelicolor* is the most effective for increasing product titers by mPKS [35]. Typical of genes from actinobacteria, *S. coelicolor* *pccA*, *pccB*, and *pccE* exhibit higher GC content than typical *A. oryzae* genes (e.g., *pccA* is 74.6% GC; *A. oryzae* ORFs have a median of 47.4% GC) and demonstrate significantly different codon bias (ESI). Therefore, *pccA*, *pccB*, and *pccE* from *S. coelicolor* were synthesized using codon-optimized DNA and the start codon was adjusted for each from GTG to ATG preferred by fungi. However, despite codon preference being optimized to *A. oryzae,* the GC% remained high at around 70%.

**Figure 3:**
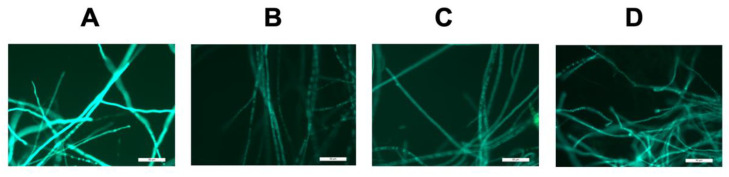
Green fluorescent imaging of: (**A**–**C**) transformants with PCC subunit genes fused to *eGFP*; (**D**) DEBS1-TE fused to eGFP. Scale bar 50 μm.

The genes encoding the PCC α, β, and ε subunits were individually cloned into fungal expression vector pTYGS·*arg* [36], under the control of the amylose-inducible *A. oryzae amyB* promoter (*P_amyB_*). In each case, these were fused in-frame at their 3′-termini with *egfp*. The resulting vectors were individually transformed into *A. oryzae* NSAR1 and after several rounds of screening on minimal media lacking arginine, selected transformants were induced in DPY media. Individual transformants were analyzed by fluorescence microscopy. Green fluorescence was detected in all cases (Figure 3), indicating that the high GC % genes are successfully transcribed and translated in *A. oryzae*. The diffuse fluorescence observed indicates that all three PCC components are expressed in the cytoplasm. However, RT-PCR for determining the expression of *pccA*, *B* and *E* was unsuccessful and we attribute this to the high GC content of the corresponding mRNA that may form complex secondary structures preventing successful amplification.

### 3.3. Heterologous Expression of DEBS1-TE in A. oryzae to form TKL

We replicated the original DEBS1-TE construction strategy^23^ *in silico* and, based on this sequence, we designed four overlapping DNA fragments spanning the 11.2 kb coding region. The four fragments were codon-optimized for *A. oryzae* and the start codon adjusted to ATG. However, like the codon-optimized *pcc* genes, the overall GC content remained high at 73%. The four fragments were re-assembled using rapid yeast recombination downstream of *P_amyB_* in pTYGS·*arg*, fused in-frame with 3′ *egfp*, and then transformed into *A. oryzae* NSAR1. The resulting transformants were inspected microscopically after induction in DPY medium and diffuse green fluorescence was detected (Figure 3D) indicative of successful cytoplasmic expression of the DEBS1-TE protein.

**Figure 4:**
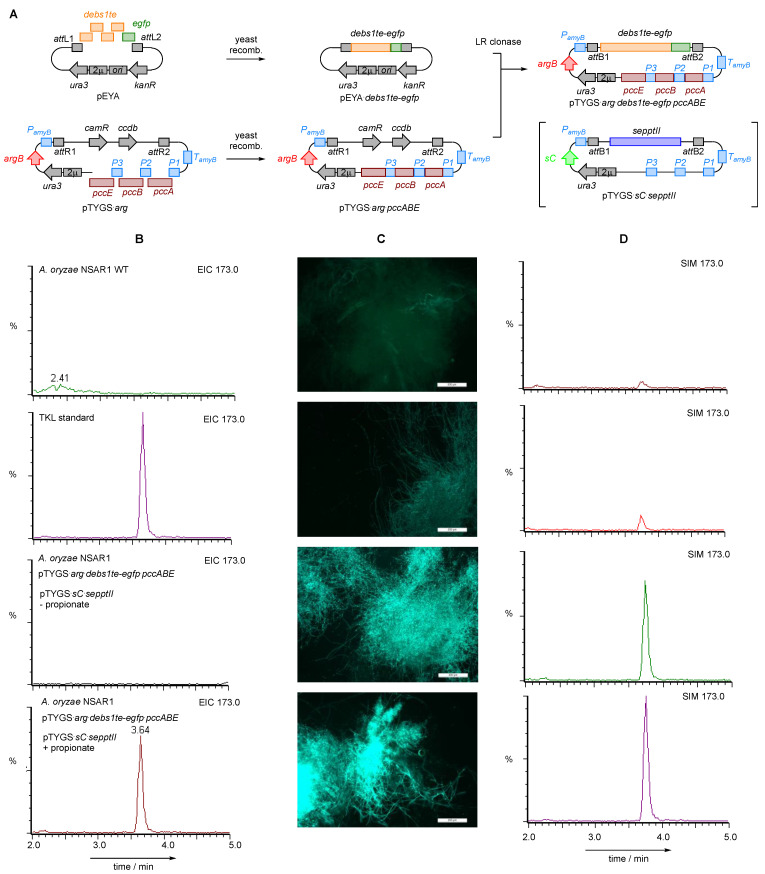
Construction of functional modular PKS in *A. oryzae*. (**A**) Construction of vectors; (**B**) LCMS analysis of *A. oryzae* NSAR1, standard TKL, and transformants in the absence and presence of propionate as indicated ES+ at *m/z* 173.0; (**C**) microscopic images of four *A. oryzae* NSAR1 transformants showing variability of eGFP intensity; (**D**) quantitative selective ion monitoring (SIM, ES+ at *m/z* 173.0) of extracts of the corresponding *A. oryzae* strains shown in panel C. Scale bars 200 μm.

We then built a single expression vector to co-express DEBS1-TE with the PCC components (Figure 4A). This time the eGFP fusions were omitted for the *pcc* components since we had established that they could be transcribed. The *debs1te-egfp* gene was placed under the control of *P_amyB_,* while the PCC components were driven by strong constitutive promoters known to be active in *A. oryzae* [36]. The transformants were cultured for 5 days and propionate (50 mM) was added the day before extraction. The cultures were extracted with ethylacetate and screened by LCMS. No new metabolites were detected in the transformant extracts (ESI). We performed single-ion monitoring for the expected mass of TKL (*m/z* 172) but no new metabolites were detected. Since it is known that DEBS1-TE is also able to accept acetate starter units we also scanned for metabolites with *m/z* = 158 but could not detect any.

We considered the most likely reason for the inactivity of the DEBS1-TE system to be the lack of phosphopantetheinylation of its three ACP domains. The *A. oryzae* PPTase NpgA homolog shares very low (7–14%) sequence identity with bacterial PPTases such as Sfp from *Bacillus subtilis* and SePptII from the erythromycin-producing strain *S. erythraea* [23,37], despite being in the same structural class (Figure S6.3) [38]. Both Sfp and SePptII are known to activate DEBS1-TE but their activities in *A. oryzae* are unknown [24,39,40].

The gene encoding SePptII from *S. erythraea* was codon optimized, synthesized, and cloned into a pTYGS vector containing an *sC* selection marker to give pTYGS·*sC*·*sepptII* [36]. The *sC* gene encodes sulfate adenyl transferase, part of the sulfur assimilation pathway of *A. oryzae*, and is normally complemented by the addition of methionine. pTYGS·*sC*·*sepptII* was then co-transformed into *A. oryzae* with pTYGS·*arg*·*pccABE*·*debs1te-egfp*. Transformants that grew on minimal media lacking arginine and methionine and that also exhibited fluorescence in inducing DPY media were selected for fermentation and chemical analysis (Figure 4). Propionate (50 mM) was added to the cultures 24 h before harvest. Cultures were extracted with ethylacetate and analyzed by LCMS. The EIC chromatogram of [M + H]^+^ 173, corresponding to TKL, showed a single new distinct peak not present in control strains. The *m/z* 173 peak was only detected when exogenous propionate was added to the culture (Figure 4B). The peak corresponded to standard synthetic TKL in terms of retention time, UV absorption, and mass fragmentation. For further clarification synthetic TKL was mixed 1:1 with the crude extract of a transformant and analyzed by LCMS, confirming precise co-elution (see ESI). Examination of randomly selected cotransformants showed a variation in the intensity of the detected eGFP fluorescence (Figure 4C). Quantitative analysis of the 173 peak for these transformants using selected ion monitoring (SIM) showed that increased fluorescence is correlated with increased production of TKL (Figure 4C,D). Synthetic TKL was then used to generate a calibration curve (see ESI) and TKL production was quantified at 0.6 mg·L^−1^ under these conditions from the most productive transformant.

### 3.4. Improving Titers of TKL through Metabolic Engineering of A. oryzae Primary Metabolism and Sodium Propionate Feeding Methods

**Figure 5:**
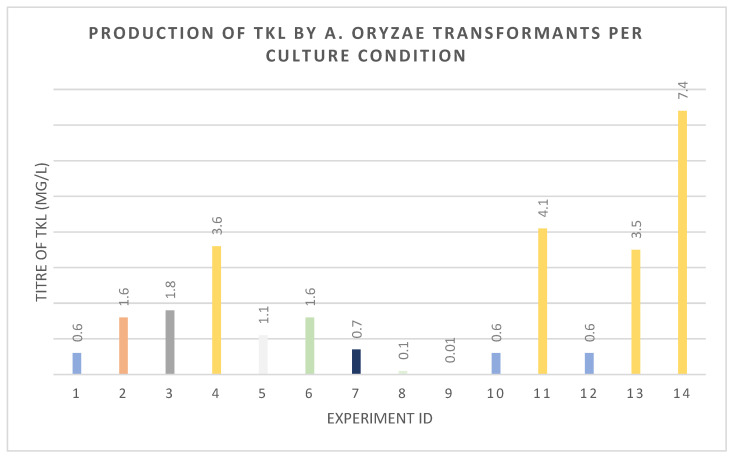
Optimization of TKL production and resulting titers of TKL. Culture condition and experiment ID described in Table 1 and Table 2.

A series of experiments were then devised in an attempt to optimize TKL production. The effects of different exogenous propionate feeding regimes were investigated for their effect on TKL production. Propionate was added at different concentrations (5–50 mM), at different time points, and in single batch-fed, pulse-fed, or continuously fed systems (Table 1 and Table 2, Figure 5). Titers of TKL were increased to 3.6 mg·L^−1^ by pulse-feeding 10 mM of sodium propionate to shake flasks at 24 h intervals over five days before extraction. Batch feeding a single high concentration of propionate or continual infusion of lower concentrations over longer periods was ineffective (Figure 5).

To determine whether the eGFP tag was interfering with the activity of DEBS1-TE, a new vector omitting eGFP was built (pTYGS·*arg*·*pccABE*·*debs1te*) and co-expressed with pTYGS·*sC*·*sepptII*. TKL titers remained at 0.6 mg·L^−1^ when strains were batch fed 50 mM propionate (Figure 5) but could be increased to 4.1 mg·L^−1^ when pulse fed with 10 mM propionate over five days.

We next wanted to determine whether propionyl-CoA synthesis was a rate-limiting factor. In an effort to ensure adequate supplies of propionyl-CoA, we over-expressed *pcsA*, encoding propionyl-CoA synthetase, while co-expressing DEBS1-TE, the PCC components, and SePptII. This was achieved by inserting a genomic copy of *A. oryzae pcsA* into the vector pTYGS·*sC·sepptII* under the control of a constitutive *Aspergillus* promoter. However, TKL titers remained at 0.6 mg·L^−1^ when strains were batch fed 50 mM propionate (Figure 5). Titers of TKL could be increased to 3.5 mg·L^−1^ through pulse feeding propionate at 10 mM over five days (Figure 5).

The MCS pathway is proposed as being the major pathway for the degradation of propionyl-CoA in fungi [41]. We inactivated *mcsA* in *A. oryzae* NSAR1 using the bipartite gene knockout strategy [42] by inserting *argB* as the selection marker into the target gene. Transformants were selected on minimal media lacking arginine and disruption was confirmed by PCR. The *mcsA* mutants were investigated for sensitivity to propionate. Compared to *A. oryzae* NSAR1, *mscA* mutants exhibited increased sensitivity to propionate, with observable growth inhibition at concentrations as low as 5 mM (Figure 2E).

We then co-transformed pTYGS·*argB·pccABE·debs1te-egfp* and pTYGS·*sC·sepptII* into the *mcsA* mutant using *sC* selection and screened for eGFP fluorescence. TKL production remained low under all feeding regimes, except when propionate was pulse-fed (5 × 10 mM aliquots, Figure 5). Under these conditions, the titer was raised to 7.4 mg·L^−1^. In a separate series of experiments, we also knocked out *coaT* in *A. oryzae* NSAR1 using the same methods. In this case, the KO strains did not exhibit a significantly different response to exogenous propionate as the *A. oryzae* NSAR control strain. Attempts were made to transform *A. oryzae* Δ*coaT* with pTYGS·*argB·pccABE·debs1te-egfp* and pTYGS·*sC·sepptII,* but transformants could not be prepared. We also attempted a double knockout to disrupt both *coaT* and *mscA* using either mutant strain as the parent strain. However, we were unable to produce the desired double mutants.

## 4. Discussion

*A. oryzae* is a highly useful host organism for the heterologous expression and engineering of fungal secondary metabolites. In particular, it has been extensively used for the production of fungal metabolites stemming from iterative type I PKS that use predominantly acetyl-CoA and malonyl-CoA for their construction [17]. Bacterial modular PKS, on the other hand, often use propionyl-CoA and methylmalonyl-CoA for the construction of polyketides [23,24,25,26,27]. The fact that propionyl-CoA is toxic to fungi, and that methylmalonyl-CoA pathways do not exist in fungi, may explain why modular PKS systems are almost unknown in these organisms.

Our results show that propionate induces PcsA and McsA in *A. oryzae*, consistent with the creation and degradation of propionyl-CoA *via* the methyl citrate cycle. The broadly selective CoaT system also seems to be constitutively active, but this system does not actually degrade the carbon skeleton of propionate and is therefore a less effective resistance mechanism. However, we assume that propionyl-CoA can be produced as a potential feedstock for modular PKS activity.

Experiments in which we fused the *S. coelicolor* PCC genes and the *S.* *erythraea* *debs1te* to *egfp* showed that all systems produced apparently full-length proteins in *A. oryzae*, showing that bacterial genes with GC% as high as 70% can be successfully transcribed and translated. Although the genes were codon optimized to *A. oryzae*, they were, for the most part, unevenly skewed towards the highest GC content codons. This adversely affected our ability to confirm transcription by RT-PCR and therefore may indicate that translation of DEBS1-TE is still inefficient. Heterologous production of non-fungal genes in *A. oryzae* has determined that codon optimization increases steady-state mRNA levels [43]. Even so, translation was possible as evidenced by GFP detection and TKL production. It will be interesting to observe whether native actinobacterial sequences will also be successfully transcribed and translated in *A. oryzae*.

Removal of the *egfp* tags and expression of *debs1te* with *pccA*, *pccB* and *pccE*, however, was not sufficient for the production of TKL in *A. oryzae*. It is common to observe that the post-translational modification of PKS ACP domains is inefficient in heterologous hosts [44]. We, therefore, co-expressed the bacterial PPTase SePptII in parallel. Under these conditions, we observed effective TKL production. Compared to other hosts used to express mPKS that lack the key acyl-CoA metabolic pathways, *A. oryzae* performs better in synthesizing TKL [39,45,46]. (Scheme 2 and Figure 5). Considering that DEBS1-TE is a chimeric mPKS, possibly lacking correct protein-protein interactions at the fusion site, we may expect to see improved titers for native mPKS systems, as observed when all three DEBS proteins were expressed in engineered *E. coli* [24].

Experiments in which all genes were fused with *egfp* indicate that DEBS1-TE and the PCC components are present in the cytoplasm of *A. oryzae*, as is PcsA [31], suggesting that propionyl-CoA should be directly available to DEBS1-TE and PCC. In contrast, studies by Brock et al. have determined that the propionyl-CoA degradation pathways are confined to the mitochondria. The MCS degradation pathway has only been functionally investigated in a few species of fungi including *Aspergillus* sp. [47], *Fusarium* sp. [48], *Cochliobolus* sp., *Trichoderma atroviride* [49], *Gibberella zeae* [50], and *Pyricularia oryzae* [51]. Here, we report the first attempts to engineer the MCS pathway with the specific aim of enabling high concentrations of propionyl-CoA for use by DEBS1-TE and PCC. Knockout of *mcsA* led to strains that were more sensitive to propionate but could produce higher titers of TKL (up to 7.4 mg·L^−1^) under pulse-feeding conditions. These results suggest that the MCS pathway is probably the major route for the degradation of propionyl CoA in *A. oryzae*.

A recent proteomic study of propionyl-CoA metabolism in the pathogenic fungus *Paracoccidioides lutzii* demonstrated that a carnitine *O*-acetyltransferase (PAAG_06224) is upregulated in response to propionate [52]. This study also identified an acetate kinase (PAAG_07180), that was only detected when *P. lutzii* was grown on propionate, which is homologous to propionate kinases in bacteria. In the bacterium *Neisseria meningitidis*, propionate kinases generate propionyl phosphate that is proposed to be converted to propionyl-CoA via phosphotransacetylase [53]. *A. oryzae* NSAR1 has homologs of both the carnitine *O*-acetyltransferase (XP_001821862.3) and putative propionate kinase (XP_02309003) with 71% and 59% similarity, respectively. Additionally, *Candida albicans* has been shown to use a modified β-oxidation pathway to degrade propionyl-CoA to acetyl-CoA [54]. Future work, therefore, could focus on characterizing these hypothetical propionyl-CoA metabolic pathways in *A. oryzae* to provide adequate substrates for PCC and DEBS1-TE.

Continuous infusion of propionate does not result in increased titers of TKL, presumably because low propionyl CoA concentrations do not provide adequate methylmalonyl-CoA for TKL construction because propionate is cleared from the system quickly by McsA and CoaT. Similarly, feeding propionate in a single batch is not effective, presumably because propionyl-CoA concentrations do not rise high enough for a long enough period. However, pulse feeding of several high concentrations of propionate consistently gave the highest titers of TKL, particularly in Δ*mcsA* strains. Our interpretation is that high concentrations of propionate temporarily overwhelm the ability of the cells to degrade propionyl-CoA, allowing a brief temporal window when PCC can create sufficient 2*S*-methylmalonyl CoA for TKL synthesis. This suggests that the system could be more productive if elevated 2*S*-methylmalonyl CoA concentrations could be maintained over longer periods. Future investigations will examine the production of this metabolite *via* isomerization of succinyl-CoA by methylmalonyl-CoA mutase. This enzyme creates 2*R*-methylmalonyl-CoA, but rapid epimerization can be catalyzed by methylmalonyl-CoA epimerase and has previously been used in *E. coli* for this purpose [55]. This system could therefore bypass the requirement for high propionate concentrations and provide 2*S*-methylmalonyl-CoA more consistently.

Recombination of synthetic gene fragments in yeast was used to rapidly build the 11.2 kb DEBS1-TE. We previously demonstrated that yeast recombination offers an excellent method for the reconstruction of large modular synthetases (e.g., a five module non-ribosomal peptide synthetase, NRPS) [56] as well as being ideally suited to the precise engineering of PKS. For example, we have achieved precise swaps of individual 12-residue protein helices in the tenellin fungal PKS-NRPS to reprogram its function [57]. Thus, there is considerable scope for the future precise engineering of mPKS using a combination of synthetic DNA and yeast recombination to achieve their rational reprogramming.

There are very few reports on PPTases in fungi and, so far, include those involved in lysine biosynthesis in yeasts, e.g., Lys5p/Lys7p, or those involved in both lysine biosynthesis and polyketide synthesis in filamentous fungi, i.e., CfwA/NpgA [58], and PPT1 from various species [59,60,61,62,63]. These PPTases have been investigated in terms of their roles in primary and secondary metabolism and the effects on fungal virulence. Here, we determined that the Sfp-type PPTase in *A. oryzae* is unable to post-translationally modify the ACP domains of DEBS1-TE. This is somewhat surprising considering that iPKS and mPKS are believed to have evolved from the same ancestor [64]. *A. oryzae* has been used as a host for heterologous expression of PKS from the lichen-forming fungus *Cladonia uncialis* but no polyketide products were detected despite correctly spliced mRNA being detected, even when codon-optimized PKS genes were utilized [65]. It was proposed that *A. oryzae*′s native PPTase is unable to post-translationally modify PKS from lichens and our investigation with DEBS1-TE would support that. Therefore, for developing any heterologous host, understanding the limitations of the native PPTase and identifying functional alternatives should not be underestimated.

In conclusion, we have metabolically engineered the fungus *A. oryzae* NSAR1 and, for the first time, show that it is capable of expressing a bacterial mPKS for polyketide production using minimal modifications of its metabolism. This approach required the introduction of the non-native propionyl-CoA carboxylase enzyme complex from *S. coelicolor* and the *S*. *erythraea* PPTase SePptII. Increased titers of TKL were observed in *mcsA* KO strains, but this modification is not essential. *A. oryzae* is easily able to express actinobacterial components for polyketide production and there seems to be no fundamental reason why native filamentous fungi cannot exploit modular PKS systems—making their absence from fungi all the more mysterious. Our work shows that the expression and engineering of bacterial components in a fungal host is simple, rapid, and effective and should offer the opportunity to create new hybrid fungal–bacterial biosynthetic pathways in the future.

## Figures and Tables

**Table 1:** Description of sodium propionate feeding regimes.

	Time after Inoculation (Days)/Sodium Propionate Inoculation Concentration (mM)										
Condition	1	2	3	4	5	6	7	8	9	10	11
A	-	-	-	50	Extract						
B	12.5	12.5	12.5	12.5	Extract						
C	50 mM infusion with pump				Extract						
D	10	10	10	10	10	Extract					
E	50 mM infusion with pump					Extract					
F	6.25	6.25	6.25	6.25	6.25	6.25	6.25	6.25	Extract		
G	5	5	5	5	5	5	5	5	5	5	Extract
H	2	2	2	2	2	Extract					
I	10 mM infusion with pump					Extract					

**Table 2:** Description of different feeding experiments perfomed on various *A. oryzae* host strains.

Experiment ID	*A. oryzae* Host	Expression Vectors	Condition
1	NSAR1	pTYGS-*arg-pccABE-debs1te-egfp* + pTYGS-*sC-sepptII*	A
2			B
3			C
4			D
5			E
6			F
7			G
8			H
9			I
10	NSAR1	pTYGS-*arg-pccABE-debs1te* + pTYGS-*sC-sepptII*	A
11			D
12	NSAR1	pTYGS-*arg-pccABE-debs1te-egfp* + pTYGS-*sC-sepptII-pcsA*	A
13			D
14	NSAR1 Δ*mcsA*	pTYGS-*arg-pccABE-debs1te-egfp* + pTYGS-*sC-sepptII*	D

## Supplementary Materials

The following are available online at https://www.mdpi.com/article/10.3390/jof7121085/s1: RT-PCR study of expression of *pcsA*, *mcsA* and *coaT* in *A. oryzae* NSAR1; codon optimization details for DEBS1-TE and PCC; sequences of DEBS1-TE and PCC; DEBS1-TE expression cassette construction; gene expression and identification of DEBS1-TE and PCC; fungal and bacterial PPTase alignment; synthesis and characterisation of of TKL; quantification of TKL; deletion of propionyl-CoA degradation pathways; deyaols of plasmids and primers used in the study.

## References

[1] Newman D.J., Cragg G.M. (2016). Natural products as sources of new drugs from 1981 to 2014. J. Nat. Prod..

[2] Wu J., Zhang Q., Deng W., Qian J., Zhang S., Liu W. (2011). Toward Improvement of Erythromycin A Production in an IndustrialSaccharopolyspora erythraea Strain via Facilitation of Genetic Manipulation with an Artificial attB Site for Specific Recombination. Appl. Environ. Microbiol..

[3] Miyazawa T., Fitzgerald B.J., Keatinge-Clay A.T. (2021). Preparative production of an enantiomeric pair by engineered polyketide synthases. Chem. Commun..

[4] Cook T.B., Pfleger B.F. (2019). Leveraging synthetic biology for producing bioactive polyketides and non-ribosomal peptides in bacterial heterologous hosts. MedChemComm.

[5] Yang D., Park S.Y., Park Y.S., Eun H., Lee S.Y. (2020). Metabolic Engineering of Escherichia coli for Natural Product Biosynthesis. Trends Biotechnol..

[6] Bond C., Tang Y., Li L. (2016). Saccharomyces cerevisiae as a tool for mining, studying and engineering fungal polyketide synthases. Fungal Genet. Biol..

[7] Harvey C.J.B., Tang M., Schlecht U., Horecka J., Fischer C.R., Lin H.-C., Li J., Naughton B., Cherry J., Miranda M. (2018). HEx: A heterologous expression platform for the discovery of fungal natural products. Sci. Adv..

[8] Xu X., Liu Y., Du G., Ledesma-Amaro R., Liu L. (2020). Microbial Chassis Development for Natural Product Biosynthesis. Trends Biotechnol..

[9] Daba G.M., Mostafa F.A., Elkhateeb W.A. (2021). The ancient koji mold (*Aspergillus oryzae*) as a modern biotechnological tool. Bioresour. Bioprocess..

[10] He Y., Cox R.J. (2016). The molecular steps of citrinin biosynthesis in fungi. Chem. Sci..

[11] Williams K., Szwalbe A.J., Mulholland N.P., Vincent J.L., Bailey A.M., Willis C.L., Simpson T.J., Cox R.J. (2016). Heterologous Production of Fungal Maleidrides Reveals the Cryptic Cyclization Involved in their Biosynthesis. Angew. Chem. Int. Ed..

[12] Kahlert L., Bassiony E.F., Cox R., Skellam E.J. (2020). Diels–Alder Reactions during the Biosynthesis of Sorbicillinoids. Angew. Chem. Int. Ed..

[13] Kahlert L., Villanueva M., Cox R.J., Skellam E.J. (2021). Biosynthesis of 6-Hydroxymellein Requires a Collaborating Polyketide Synthase-like Enzyme. Angew. Chem. Int. Ed..

[14] Feng J., Surup F., Hauser M., Miller A., Wennrich J.-P., Stadler M., Cox R.J., Kuhnert E. (2020). Biosynthesis of oxygenated brasilane terpene glycosides involves a promiscuous *N*-acetylglucosamine transferase. Chem. Commun..

[15] Schor R., Schotte C., Wibberg D., Kalinowski J., Cox R.J. (2018). Three previously unrecognised classes of biosynthetic enzymes revealed during the production of xenovulene A. Nat. Commun..

[16] Nofiani R., De Mattos-Shipley K., Lebe K.E., Han L.-C., Iqbal Z., Bailey A., Willis C.L., Simpson T.J., Cox R.J. (2018). Strobilurin biosynthesis in Basidiomycete fungi. Nat. Commun..

[17] Kahlert L., Schotte C., Cox R.J. (2021). Total Mycosynthesis: Rational Bioconstruction and Bioengineering of Fungal Natural Products. Synthesis.

[18] Alberti F., Khairudin K., Venegas E.R., Davies J., Hayes P.M., Willis C.L., Bailey A.M., Foster G.D. (2017). Heterologous expression reveals the biosynthesis of the antibiotic pleuromutilin and generates bioactive semi-synthetic derivatives. Nat. Commun..

[19] Bailey A., Alberti F., Kilaru S., Collins C., De Mattos-Shipley K., Hartley A.J., Hayes P., Griffin A., Lazarus C.M., Cox R. (2016). Identification and manipulation of the pleuromutilin gene cluster from Clitopilus passeckerianus for increased rapid antibiotic production. Sci. Rep..

[20] Rendsvig J.K.H., Futyma M.E., Jarczynska Z.D., Mortensen U.H., Benz J.P., Schipper K. (2020). Filamentous Fungi as Hosts for Heterologous Production of Proteins and Secondary Metabolites in the Post-Genomic Era. Genetics and Biotechnology.

[21] Thynne E., Mead O., Chooi Y.-H., McDonald M.C., Solomon P.S. (2019). Acquisition and Loss of Secondary Metabolites Shaped the Evolutionary Path of Three Emerging Phytopathogens of Wheat. Genome Biol. Evol..

[22] Cox R. (2014). Oxidative rearrangements during fungal biosynthesis. Nat. Prod. Rep..

[23] Cortes J., Wiesmann K.E.H., Roberts G.A., Brown M.J.B., Staunton J., Leadlay P.F. (1995). Repositioning of a Domain in a Modular Polyketide Synthase to Promote Specific Chain Cleavage. Science.

[24] Pfeifer B.A., Admiraal S.J., Gramajo H., Cane D.E., Khosla C. (2001). Biosynthesis of Complex Polyketides in a Metabolically Engineered Strain of *E. coli*. Science.

[25] Kao C.M., Luo G., Katz L., Cane D.E., Khosla C. (1995). Manipulation of macrolide ring size by directed mutagenesis of a modular polyketide synthase. J. Am. Chem. Soc..

[26] Kim B.S., Cropp T.A., Florova G., Lindsay Y., Sherman D.H., Reynolds K.A. (2002). An Unexpected Interaction between the Modular Polyketide Synthases, Erythromycin DEBS1 and Pikromycin PikAIV, Leads to Efficient Triketide Lactone Synthesis. Biochemistry.

[27] Pieper R., Luo G., Cane D.E., Khosla C. (1995). Cell-free synthesis of polyketides by recombinant erythromycin polyketide synthases. Nat. Cell Biol..

[28] Brock M., Buckel W. (2004). On the mechanism of action of the antifungal agent propionate. JBIC J. Biol. Inorg. Chem..

[29] Zhang Y.-Q., Keller N.P. (2004). Blockage of methylcitrate cycle inhibits polyketide production in Aspergillus nidulans. Mol. Microbiol..

[30] Lazarus C.M., Williams K., Bailey A.M. (2014). Reconstructing fungal natural product biosynthetic pathways. Nat. Prod. Rep..

[31] Zhang Y.-Q., Brock M., Keller N.P. (2004). Connection of Propionyl-CoA Metabolism to Polyketide Biosynthesis in Aspergillus nidulans. Genetics.

[32] Brock M., Fischer R., Linder D., Buckel W. (2000). Methylcitrate synthase from Aspergillus nidulans: Implications for propionate as an antifungal agent. Mol. Microbiol..

[33] Fleck C.B., Brock M. (2008). Characterization of an acyl-CoA: Carboxylate CoA-transferase from Aspergillus nidulans involved in propionyl-CoA detoxification. Mol. Microbiol..

[34] Diacovich L., Peirú S., Kurth D., Rodríguez E., Podestá F., Khosla C., Gramajo H. (2002). Kinetic and Structural Analysis of a New Group of Acyl-CoA Carboxylases Found in Streptomyces coelicolor A3 (2). J. Biol. Chem..

[35] Vandova G.A., O’Brien R.V., Lowry B., Robbins T.F., Fischer C.R., Davis R.W., Khosla C., Harvey C.J.B., Hillenmeyer M.E. (2017). Heterologous expression of diverse propionyl-CoA carboxylases affects polyketide production in *Escherichia coli*. J. Antibiot..

[36] Pahirulzaman K.A.K., Williams K., Lazarus C.M. (2012). A Toolkit for Heterologous Expression of Metabolic Pathways in *Aspergillus oryzae*. Methods Enzymol..

[37] Lambalot R.H., Gehring A.M., Flugel R.S., Zuber P., LaCelle M., Marahiel M.A., Reid R., Khosla C., Walsh C.T. (1996). A new enzyme superfamily—the phosphopantetheinyl transferases. Chem. Biol..

[38] Kim J.H., Komatsu M., Shin-ya K., Omura S., Ikeda H. (2018). Distribution and functional analysis of the phosphopantetheinyl transferase superfamily in Actinomycetales microorganisms. Proc. Nat. Acad. Sci. USA.

[39] Mutka S.C., Bondi S.M., Carney J.R., Da Silva N.A., Kealey J.T. (2006). Metabolic pathway engineering for complex polyketide biosynthesis inSaccharomyces cerevisiae. FEMS Yeast Res..

[40] Weissman K.J., Hong H., Oliynyk M., Siskos A.P., Leadlay P.F. (2004). Identification of a Phosphopantetheinyl Transferase for Erythromycin Biosynthesis in *Saccharopolyspora erythraea*. ChemBioChem.

[41] Brock M., Timmis K.N. (2010). Role of Cellular Control of Propionyl-CoA Levels for Microbial Pathogenesis. Handbook of Hydrocarbon and Lipid Microbiology.

[42] Nielsen M.L., Albertsen L., Lettier G., Nielsen J.B., Mortensen U.H. (2006). Efficient PCR-based gene targeting with a recyclable marker for *Aspergillus nidulans*. Fungal Genet. Biol..

[43] Tokuoka M., Tanaka M., Ono K., Takagi S., Shintani T., Gomi K. (2008). Codon Optimization Increases Steady-State mRNA Levels in Aspergillus oryzae Heterologous Gene Expression. Appl. Environ. Microbiol..

[44] Cox R.J., Hitchman T.S., Byrom K.J., Findlow I.C., A Tanner J., Crosby J., Simpson T.J. (1997). Post-translational modification of heterologously expressed Streptomyces type II polyketide synthase acyl carrier proteins. FEBS Lett..

[45] Gonzalez-Garcia R.A., Nielsen L.K., Marcellin E. (2020). Heterologous Production of 6-Deoxyerythronolide B in *Escherichia coli* through the Wood Werkman Cycle. Metabolites.

[46] Zhang H., Boghigian B.A., Pfeifer B.A. (2010). Investigating the role of native propionyl-CoA and methylmalonyl-CoA metabolism on heterologous polyketide production in *Escherichia coli*. Biotech. Bioeng..

[47] Maerker C., Rohde M., Brakhage A.A., Brock M. (2005). Methylcitrate synthase from *Aspergillus fumigatus*. Propionyl-CoA affects polyketide synthesis, growth and morphology of conidia. FEBS J..

[48] Domin N., Wilson D., Brock M. (2009). Methylcitrate cycle activation during adaptation of Fusarium solani and Fusarium verticillioides to propionyl-CoA-generating carbon sources. Microbiology.

[49] Dubey M.K., Broberg A., Jensen D., Karlsson M. (2013). Role of the methylcitrate cycle in growth, antagonism and induction of systemic defence responses in the fungal biocontrol agent *Trichoderma atroviride*. Microbiology.

[50] Lee S.-H., Han Y.-K., Yun S.-H., Lee Y.-W. (2009). Roles of the Glyoxylate and Methylcitrate Cycles in Sexual Development and Virulence in the Cereal Pathogen *Gibberella zeae*. Eukaryot. Cell.

[51] Yan Y., Wang H., Zhu S., Wang J., Liu X., Lin F., Lu J. (2019). The Methylcitrate Cycle is Required for Development and Virulence in the Rice Blast Fungus *Pyricularia oryzae*. Mol. Plant-Microbe Interact..

[52] Santos L.P.A., Assunção L.D.P., Lima P.D.S., Tristão G.B., Brock M., Borges C.L., Silva-Bailao M., Soares C.M.D.A., Bailao A. (2020). Propionate metabolism in a human pathogenic fungus: Proteomic and biochemical analyses. IMA Fungus.

[53] Catenazzi M.C.E., Jones H., Wallace I., Clifton J., Chong J.P.J., Jackson M.A., Macdonald S., Edwards J., Moir J.W.B. (2014). A large genomic island allows Neisseria meningitidis to utilize propionic acid, with implications for colonization of the human nasopharynx. Mol. Microbiol..

[54] Otzen C., Bardl B., Jacobsen I.D., Nett M., Brock M. (2014). Candida albicans utilizes a modified β-oxidation pathway for the degradation of toxic propionyl-CoA. J. Biol. Chem..

[55] Dayem L.C., Carney J.R., Santi D.V., Pfeifer B.A., Khosla C., Kealey J.T. (2002). Metabolic engineering of a methylmalonyl-CoA mutase-epimerase pathway for complex polyketide biosynthesis in *Escherichia coli*. Biochemistry.

[56] De Mattos-Shipley K.M.J., Greco C., Heard D.M., Hough G., Mulholland N.P., Vincent J.L., Micklefield J., Simpson T.J., Willis C.L., Cox R.J. (2018). The cycloaspeptides: Uncovering a new model for methylated nonribosomal peptide biosynthesis. Chem. Sci..

[57] Yang X.-L., Friedrich S., Yin S., Piech O., Williams K., Simpson T.J., Cox R.J. (2019). Molecular basis of methylation and chain-length programming in a fungal iterative highly reducing polyketide synthase. Chem. Sci..

[58] Márquez-Fernández O., Trigos A., Ramos-Balderas J.L., Viniegra-González G., Deising H.B., Aguirre J. (2007). Phosphopantetheinyl Transferase CfwA/NpgA Is Required for Aspergillus nidulans Secondary Metabolism and Asexual Development. Eukaryot. Cell.

[59] Zainudin N.A.I.M., Condon B., De Bruyne L., Van Poucke C., Bi Q., Li W., Höfte M., Turgeon B.G. (2015). Virulence, Host-Selective Toxin Production, and Development of Three Cochliobolus Phytopathogens Lacking the Sfp-Type 4′-Phosphopantetheinyl Transferase Ppt1. Mol. Plant-Microbe Interact..

[60] Albermann S., Elter T., Teubner A., Krischke W., Hirth T., Tudzynski B. (2013). Characterization of novel mutants with an altered gibberellin spectrum in comparison to different wild-type strains of *Fusarium fujikuroi*. Appl. Microbiol. Biotechnol..

[61] Leng Y., Zhong S. (2012). Sfp-type 4′-phosphopantetheinyl transferase is required for lysine synthesis, tolerance to oxidative stress and virulence in the plant pathogenic fungus *Cochliobolus sativus*. Mol. Plant Pathol..

[62] Velazquez-Robledo R., Contreras-Cornejo H.A., Macias-Rodriguez L., Hernandez-Morales A., Aguirre J., Casa-Flores S., Lopez-Bucio J., Herrera-Estrella A. (2011). Role of the 4-Phosphopantetheinyl Transferase of *Trichoderma virens* in Secondary Metabolism and Induction of Plant Defense Responses. MPMI.

[63] Horbach R., Graf A., Weihmann F., Antelo L., Mathea S., Liermann J.C., Opatz T., Thines E., Aguirre J., Deising H.B. (2009). Sfp-Type 4′-Phosphopantetheinyl Transferase Is Indispensable for Fungal Pathogenicity. Plant Cell.

[64] Jenke-Kodama H., Sandmann A., Müller R., Dittmann E. (2005). Evolutionary Implications of Bacterial Polyketide Synthases. Mol. Biol. Evol..

[65] Bertrand R.L., Sorensen J.L. (2019). Lost in Translation: Challenges with Heterologous Expression of Lichen Polyketide Synthases. Chem. Sel..

