# Supplementary material for: Engineering Aspergillus oryzae for the Heterologous Expression of a Bacterial Modular Polyketide Synthase
Source: J Fungi (Basel). 2021 Dec 17;7(12):1085. doi: 10.3390/jof7121085 (PMC8708903; doi:10.3390/jof7121085)
Supplement: Supplementary file 1 [file jof-07-01085-s001.zip › jof-1503728-supplementary.pdf]

Engineering *Aspergillus oryzae* for the Heterologous Expression of a Bacterial Modular Polyketide Synthase

Jin Feng,[a] Maurice Hauser,[a] Russell J. Cox[a*] and Elizabeth Skellam[a,b*]

[a] Institute for Organic Chemistry and Biomolekular Wirkstoff Zentrum, Leibniz University Hannover, Schneiderberg 38, Hannover, 30167, Germany.

[b] Department of Chemistry and BioDiscovery Institute, University of North Texas,
* Elizabeth.skellam@unt.edu, Russell.cox@oci.uni-hannover.de;

Electronic Supplementary Information

Contents

1. RT-PCR Study of Expression of *pcsA*, *mcsA* and *coaT* in *A. oryzae* NSAR1
2. Codon Optimization for DEBS1-TE and PCC
3. Sequences of DEBS1-TE and PCC
4. DEBS1-TE Expression Cassette Construction
5. Gene Expression Identification of DEBS1-TE and PCC
6. Fungal and Bacterial PPTase Alignment
7. Synthesis of TKL
8. Quantification of TKL
9. Deletion of Propionyl-CoA Degradation Pathways
10. Plasmids and Primers
11. References

1. RT-PCR Study of Expression of *pcsA*, *mcsA* and *coaT* in *A. oryzae* NSAR1

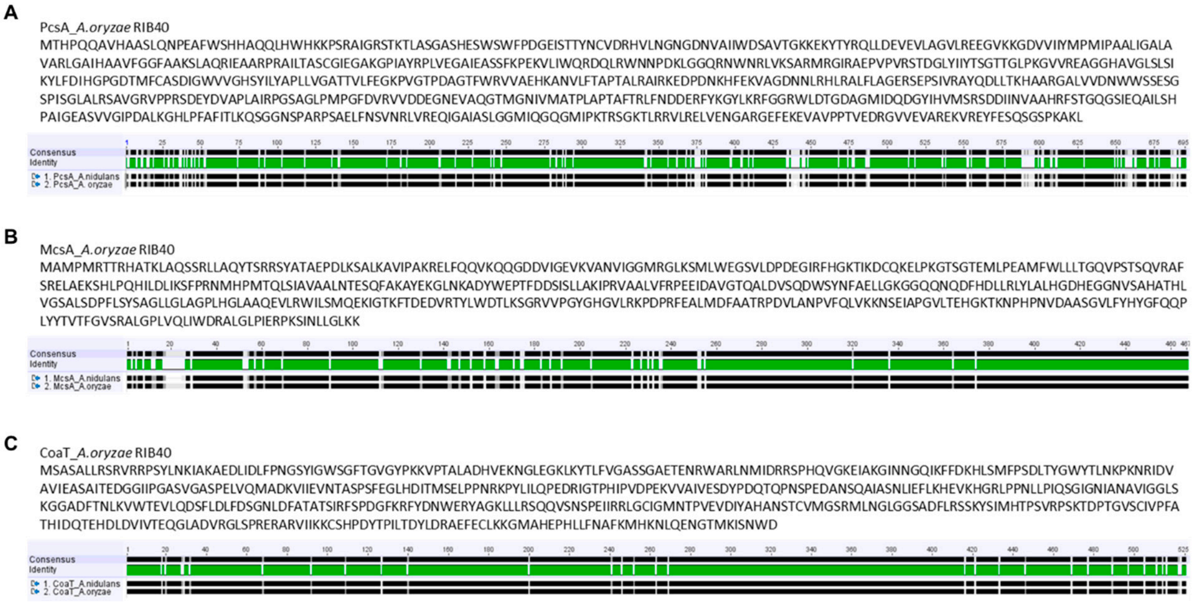

Figure S1.1 Alignment of protein sequences of propionyl-CoA synthetase (PcsA), methylcitrate synthase (McsA), and CoA-transferase (CoaT): **A**, PcsA alignment of *A. nidulans* PcsA (accession number AY102074) and *A. oryzae* PcsA (accession number NC_036441); **B**, McsA alignment of *A. nidulans* McsA (accession number AJ249117) and *A. oryzae* McsA (accession number XP_001817006); **C**, CoaT alignment of *A. nidulans* CoaT (accession number XP_659151) and *A. oryzae* CoaT (accession number XP_001817633).

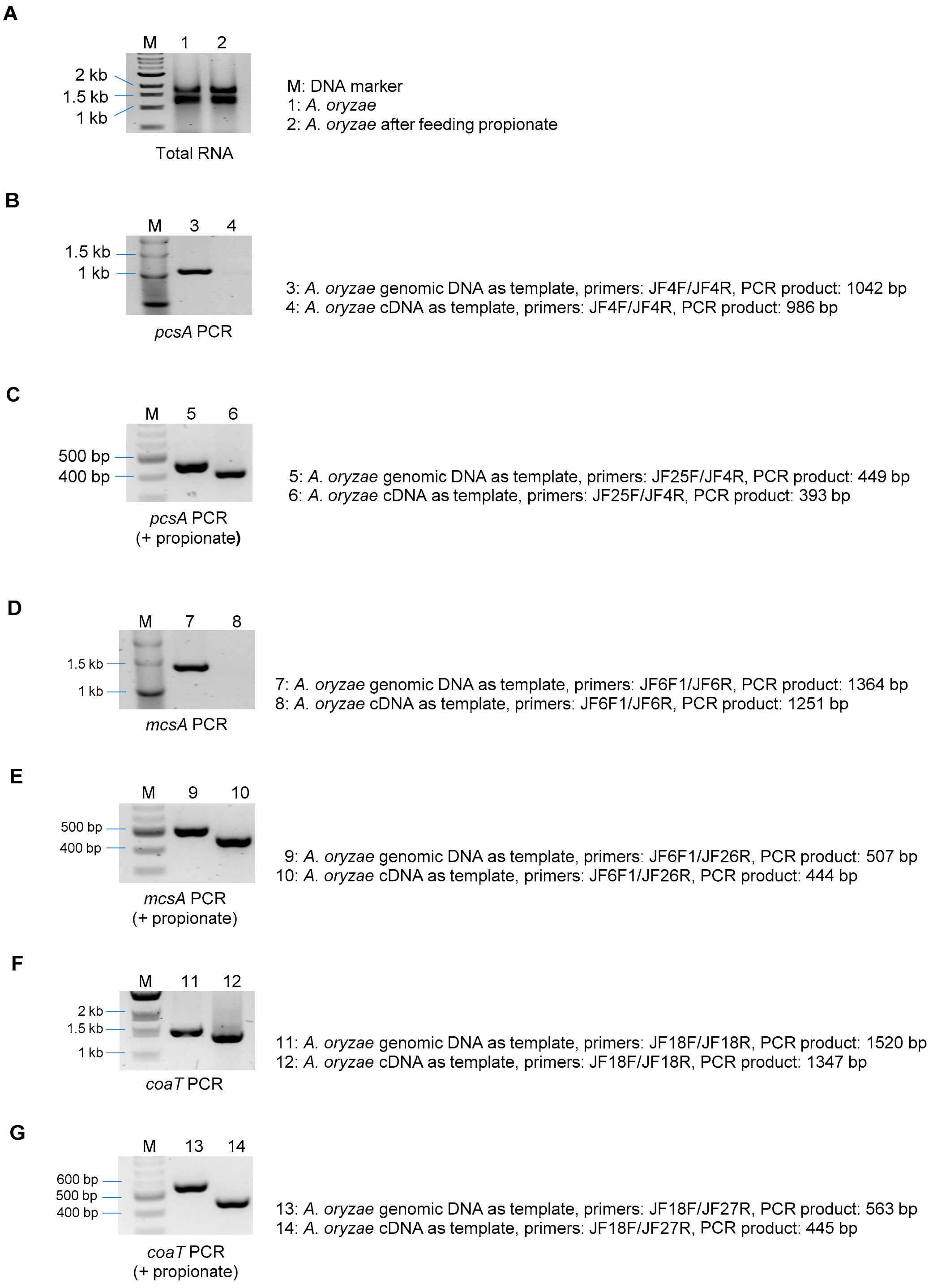

Figure S1.2 RT-PCR identification of gene expression of *pcsA*, *mcsA* and *coaT* in *A. oryzae* NSAR1.

2. Codon Optimization for DEBS1-TE and PCC

Table S1 Comparison of codon usage in *A. oryzae* genes with that in native and codon-optimized DEBS1-TE.

Amino acid	Codon	Frequency (%)			Amino acid	Codon	Frequency (%)		
		A. oryzae	DEBS1-TE				*A. oryzae*	DEBS1-TE	
			Native	Optimized				Native	Optimized
Ala	GCG	20	50	0	Leu	CTT	19	3	0
	GCA	23	3	0		CTC	23	31	100
	GCT	27	2	0		CTA	11	0	0
	GCC	31	45	100		CTG	22	59	0
Arg	CGT	18	6	0	Lys	AAA	36	0	8
	CGC	23	48	100		AAG	64	0	92
	CGA	17	4	0	Met	ATG	100	100	100
	CGG	18	36	0	Phe	TTT	38	1	0
	AGA	13	0	0		TTC	62	99	100
	AGG	12	6	0	Pro	CCT	27	4	100
Asn	AAT	45	8	0		CCC	26	35	0
	AAC	55	92	100		CCA	25	2	0
Asp	GAT	53	5	100		CCG	22	59	0
	GAC	47	95	0	Ser	AGT	13	2	0
Cys	TGT	46	10	0		AGC	18	22	0
	TGC	54	90	100		TCT	18	1	0
Gln	CAA	43	7	0		TCC	20	27	100
	CAG	57	93	100		TCA	14	2	0
Glu	GAA	44	23	0		TCG	16	46	0
	GAG	56	77	100	Thr	ACT	24	3	0
Gly	GGT	28	19	0		ACC	32	60	100
	GGC	31	52	100		ACA	24	1	0
	GGA	24	9	0		ACG	20	36	0
	GGG	17	20	0	Trp	TGG	100	100	100
His	CAT	47	7	100	Tyr	TAT	47	5	0
	CAC	53	93	0		TAC	53	95	100
Ile	ATT	36	1	0	Val	GTT	27	4	0
	ATC	50	97	100		GTC	33	44	100
	ATA	14	2	0		GTA	13	0	0
Leu	TTA	7	0	0		GTG	27	52	0
	TTG	18	7	0					

0 50 100

Table S2 Comparison of codon usage in *A. oryzae* genes with that in native and codon-optimized *pccABE* genes.

Amino acid	Codon	Frequency (%)						
		A. oryzae	*pccA*		*pccB*		*pccE*	
			Native	Optimized	Native	Optimized	Native	Optimized
Ala	GCG	20	18	0	26	0	27	0
	GCA	23	1	0	2	0	9	0
	GCT	27	2	0	2	0	0	0
	GCC	31	78	100	71	100	64	100
Arg	CGT	18	7	0	6	0	0	0
	CGC	23	76	100	84	100	67	100
	CGA	17	0	0	0	0	0	0
	CGG	18	14	0	6	0	17	0
	AGA	13	0	0	0	0	17	0
	AGG	12	2	0	3	0	0	0
Asn	AAT	45	14	0	0	0	0	0
	AAC	55	86	100	100	100	100	100
Asp	GAT	53	6	100	3	100	0	100
	GAC	47	94	0	97	0	100	0
Cys	TGT	46	0	0	25	0	0	0
	TGC	54	100	100	75	100	0	0
Gln	CAA	43	8	0	11	0	0	0
	CAG	57	92	100	89	100	0	0
Glu	GAA	44	20	0	11	0	67	0
	GAG	56	80	100	89	100	33	100
Gly	GGT	28	5	0	8	0	0	0
	GGC	31	88	100	92	100	100	100
	GGA	24	0	0	0	0	0	0
	GGG	17	7	0	0	0	0	0
His	CAT	47	0	100	0	100	0	100
	CAC	53	100	0	100	0	100	0
Ile	ATT	36	0	0	0	0	0	0
	ATC	50	96	100	100	100	100	100
	ATA	14	4	0	0	0	0	0
Leu	TTA	7	0	0	0	0	0	0
	TTG	18	2	0	0	0	0	0
	CTT	19	0	0	0	0	0	0
	CTC	23	52	100	47	100	0	100
	CTA	11	0	0	0	0	0	0
	CTG	22	46	0	53	0	100	0
Lys	AAA	36	10	0	0	0	0	0
	AAG	64	90	100	100	100	100	100
Met	ATG	100	100	100	100	100	100	100
Phe	TTT	38	0	0	0	0	0	0
	TTC	62	100	100	100	100	0	0
Pro	CCT	27	0	100	0	100	0	100
	CCC	26	79	0	62	0	22	0
	CCA	25	0	0	0	0	0	0
	CCG	22	21	0	38	0	78	0
Ser	AGT	13	0	0	0	0	0	0
	AGC	18	10	0	9	0	0	0
	TCT	18	0	0	0	0	0	0
	TCC	20	72	100	77	100	67	100
	TCA	14	3	0	0	0	0	0
	TCG	16	14	0	14	0	33	0
Thr	ACT	24	0	0	3	0	0	0
	ACC	32	75	100	62	100	70	100
	ACA	24	0	0	0	0	0	0
	ACG	20	25	0	35	0	30	0
Trp	TGG	100	100	100	100	100	100	100
Tyr	TAT	47	13	0	0	0	0	0
	TAC	53	88	100	100	100	100	100
Val	GTT	27	0	0	0	0	0	0
	GTC	33	81	100	72	100	50	100
	GTA	13	2	0	0	0	0	0
	GTG	27	17	0	28	0	50	0

0 50 100

3. Sequences of DEBS1-TE and PCC

Codon-optimized DEBS1-TE:

ATGGCCGATCTCTCCAAGCTCTCCGATTCCCGCACCGCCCAGCCTGGCCGCATCGTCCGCCCTTGGCCTCTCTCCGGCTGCAA
CGAGTCCGCCCTCCGCGCCCGCGCCCGCCAGCTCCGCGCCCATCTCGATCGCTTCCCTGATGCCGGCGTCGAGGGCGTCGG
CGCCGCCCTCGCCCATGATGAGCAGGCCGATGCCGGCCCTCATCGCGCCGTCGTCGTCGCCTCCTCCACCTCCGAGCTCCTC
GATGGCCTCGCCGCCGTCGCCGATGGCCGCCCTCATGCCTCCGTCGTCCGCGGCGTCGCCCGCCCTTCCGCCCCTGTCGTC
TTCGTCTTCCCTGGCCAGGGCGCCCAGTGGGCCGGCATGGCCGGCGAGCTCCTCGGCGAGTCCCGCGTCTTCGCCGCCGCC
ATGGATGCCTGCGCCCGCGCCTTCGAGCCTGTCACCGATTGGACCCTCGCCCAGGTCCTCGATTCCCCTGAGCAGTCCCGCC
GCGTCGAGGTCGTCCAGCCTGCCCTCTTCGCCGTCCAGACCTCCCTCGCCGCCCTCTGGCGCTCCTTCGGCGTCACCCCTGA
TGCCGTCGTCGGCCATTCCATCGGCGAGCTCGCCGCCGCCCATGTCTGCGGCGCCGCCGGCGCCGCCGATGCCGCCCGCGC
CGCCGCCCTCTGGTCCCGCGAGATGATCCCTCTCGTCGGCAACGGCGATATGGCCGCCGTCGCCCTCTCCGCCGATGAGATC
GAGCCTCGCATCGCCCGCTGGGATGATGATGTCGTCCTCGCCGGCGTCAACGGCCCTCGCTCCGTCCTCCTCACCGGCTCCC
CTGAGCCTGTCGCCCGCCGCGTCCAGGAGCTCTCCGCCGAGGGCGTCCGCGCCCAGGTCATCAACGTCTCCATGGCCGCCC
ATTCCGCCCAGGTCGATGATATCGCCGAGGGCATGCGCTCCGCCCTCGCCTGGTTCGCCCCTGGCGGCTCCGAGGTCCCTTT
CTACGCCTCCCTCACCGGCGGCGCCGTCGATACCCGCGAGCTCGTCGCCGATTACTGGCGCCGCTCCTTCCGCCTCCCTGTC
CGCTTCGATGAGGCCATCCGCTCCGCCCTCGAGGTCGGCCCTGGCACCTTCGTCGAGGCCTCCCCTCATCCTGTCCTCGCCG
CCGCCCTCCAGCAGACCCTCGATGCCGAGGGCTCCTCCGCCGCCGTCGTCCCTACCCTCCAGCGCGGCCAGGGCGGCATGC
GCCGCTTCCTCCTCGCCGCCGCCCAGGCCTTCACCGGCGGCGTCGCCGTCGATTGGACCGCCGCCTACGATGATGTCGGCG
CCGAGCCTGGCTCCCTCCCTGAGTTCGCCCCTGCCGAGGAGGAGGATGAGCCTGCCGAGTCCGGCGTCGATTGGAACGCCC
CTCCTCATGTCCTCCGCGAGCGCCTCCTCGCCGTCGTCAACGGCGAGACCGCCGCCCTCGCCGGCCGCGAGGCCGATGCCG
AGGCCACCTTCCGCGAGCTCGGCCTCGATTCCGTCCTCGCCGCCCAGCTCCGCGCCAAGGTCTCCGCCGCCATCGGCCGCG
AGGTCAACATCGCCCTCCTCTACGATCATCCTACCCCTCGCGCCCTCGCCGAGGCCCTCGCCGCCGGCACCGAGGTCGCCCA
GCGCGAGACCCGCGCCCGCACCAACGAGGCCGCCCCTGGCGAGCCTGTCGCCGTCGTCGCCATGGCCTGCCGCCTCCCTGG
CGGCGTCTCCACCCCTGAGGAGTTCTGGGAGCTCCTCTCCGAGGGCCGCGATGCCGTCGCCGGCCTCCCTACCGATCGCGG
CTGGGATCTCGATTCCCTCTTCCATCCTGATCCTACCCGCTCCGGCACCGCCCATCAGCGCGGCGGCGGCTTCCTCACCGAG
GCCACCGCCTTCGATCCTGCCTTCTTCGGCATGTCCCCTCGCGAGGCCCTCGCCGTCGATCCTCAGCAGCGCCTCATGCTCGA
GCTCTCCTGGGAGGTCCTCGAGCGCGCCGGCATCCCTCCTACCTCCCTCCAGGCCTCCCCTACCGGCGTCTTCGTCGGCCTC
ATCCCTCAGGAGTACGGCCCTCGCCTCGCCGAGGGCGGCGAGGGCGTCGAGGGCTACCTCATGACCGGCACCACCACCTCC
GTCGCCTCCGGCCGCATCGCCTACACCCTCGGCCTCGAGGGCCCTGCCATCTCCGTCGATACCGCCTGCTCCTCCTCCCTCG
TCGCCGTCCATCTCGCCTGCCAGTCCCTCCGCCGCGGCGAGTCCTCCCTCGCCATGGCCGGCGGCGTCACCGTCATGCCTAC
CCCTGGCATGCTCGTCGATTTCTCCCGCATGAACTCCCTCGCCCCTGATGGCCGCTGCAAGGCCTTCTCCGCCGGCGCCAAC
GGCTTCGGCATGGCCGAGGGCGCCGGCATGCTCCTCCTCGAGCGCCTCTCCGATGCCCGCCGCAACGGCCATCCTGTCCTC
GCCGTCCTCCGCGGCACCGCCGTCAACTCCGATGGCGCCTCCAACGGCCTCTCCGCCCCTAACGGCCGCGCCCAGGTCCGC
GTCATCCAGCAGGCCCTCGCCGAGTCCGGCCTCGGCCCTGCCGATATCGATGCCGTCGAGGCCCATGGCACCGGCACCCGC
CTCGGCGATCCTATCGAGGCCCGCGCCCTCTTCGAGGCCTACGGCCGCGATCGCGAGCAGCCTCTCCATCTCGGCTCCGTCA
AGTCCAACCTCGGCCATACCCAGGCCGCCGCCGGCGTCGCCGGCGTCATCAAGATGGTCCTCGCCATGCGCGCCGGCACCC
TCCCTCGCACCCTCCATGCCTCCGAGCGCTCCAAGGAGATCGATTGGTCCTCCGGCGCCATCTCCCTCCTCGATGAGCCTGAG
CCTTGGCCTGCCGGCGCCCGCCCTCGCCGCGCCGGCGTCTCCTCCTTCGGCATCTCCGGCACCAACGCCCATGCCATCATCG
AGGAGGCCCCTCAGGTCGTCGAGGGCGAGCGCGTCGAGGCCGGCGATGTCGTCGCCCCTTGGGTCCTCTCCGCCTCCTCCG
CCGAGGGCCTCCGCGCCCAGGCCGCCCGCCTCGCCGCCCATCTCCGCGAGCATCCTGGCCAGGATCCTCGCGATATCGCCT
ACTCCCTCGCCACCGGCCGCGCCGCCCTCCCTCATCGCGCCGCCTTCGCCCCTGTCGATGAGTCCGCCGCCCTCCGCGTCCT
CGATGGCCTCGCCACCGGCAACGCCGATGGCGCCGCCGTCGGCACCTCCCGCGCCCAGCAGCGCGCCGTCTTCGTCTTCCC
TGGCCAGGGCTGGCAGTGGGCCGGCATGGCCGTCGATCTCCTCGATACCTCCCCTGTCTTCGCCGCCGCCCTCCGCGAGTGC
GCCGATGCCCTCGAGCCTCATCTCGATTTCGAGGTCATCCCTTTCCTCCGCGCCGAGGCCGCCCGCCGCGAGCAGGATGCCG
CCCTCTCCACCGAGCGCGTCGATGTCGTCCAGCCTGTCATGTTCGCCGTCATGGTCTCCCTCGCCTCCATGTGGCGCGCCCAT
GGCGTCGAGCCTGCCGCCGTCATCGGCCATTCCCAGGGCGAGATCGCCGCCGCCTGCGTCGCCGGCGCCCTCTCCCTCGAT
GATGCCGCCCGCGTCGTCGCCCTCCGCTCCCGCGTCATCGCCACCATGCCTGGCAACAAGGGCATGGCCTCCATCGCCGCCC
CTGCCGGCGAGGTCCGCGCCCGCATCGGCGATCGCGTCGAGATCGCCGCCGTCAACGGCCCTCGCTCCGTCGTCGTCGCCG
GCGATTCCGATGAGCTCGATCGCCTCGTCGCCTCCTGCACCACCGAGTGCATCCGCGCCAAGCGCCTCGCCGTCGATTACGC
CTCCCATTCCTCCCATGTCGAGACCATCCGCGATGCCCTCCATGCCGAGCTCGGCGAGGATTTCCATCCTCTCCCTGGCTTCG
TCCCTTTCTTCTCCACCGTCACCGGCCGCTGGACCCAGCCTGATGAGCTCGATGCCGGCTACTGGTACCGCAACCTCCGCCGC
ACCGTCCGCTTCGCCGATGCCGTCCGCGCCCTCGCCGAGCAGGGCTACCGCACCTTCCTCGAGGTCTCCGCCCATCCTATCC
TCACCGCCGCCATCGAGGAGATCGGCGATGGCTCCGGCGCCGATCTCTCCGCCATCCATTCCCTCCGCCGCGGCGATGGCTC
CCTCGCCGATTTCGGCGAGGCCCTCTCCCGCGCCTTCGCCGCCGGCGTCGCCGTCGATTGGGAGTCCGTCCATCTCGGCACC
GGCGCCCGCCGCGTCCCTCTCCCTACCTACCCTTTCCAGCGCGAGCGCGTCTGGCTCGAGCCTAAGCCTGTCGCCCGCCGCT
CCACCGAGGTCGATGAGGTCTCCGCCCTCCGCTACCGCATCGAGTGGCGCCCTACCGGCGCCGGCGAGCCTGCCCGCCTCG
ATGGCACCTGGCTCGTCGCCAAGTACGCCGGCACCGCCGATGAGACCTCCACCGCCGCCCGCGAGGCCCTCGAGTCCGCCG
GCGCCCGCGTCCGCGAGCTCGTCGTCGATGCCCGCTGCGGCCGCGATGAGCTCGCCGAGCGCCTCCGCTCCGTCGGCGAG
GTCGCCGGCGTCCTCTCCCTCCTCGCCGTCGATGAGGCCGAGCCTGAGGAGGCCCCTCTCGCCCTCGCCTCCCTCGCCGATA
CCCTCTCCCTCGTCCAGGCCATGGTCTCCGCCGAGCTCGGCTGCCCTCTCTGGACCGTCACCGAGTCCGCCGTCGCCACCGG
CCCTTTCGAGCGCGTCCGCAACGCCGCCCATGGCGCCCTCTGGGGCGTCGGCCGCGTCATCGCCCTCGAGAACCCTGCCGT
CTGGGGCGGCCTCGTCGATGTCCCTGCCGGCTCCGTCGCCGAGCTCGCCCGCCATCTCGCCGCCGTCGTCTCCGGCGGCGC
CGGCGAGGATCAGCTCGCCCTCCGCGCCGATGGCGTCTACGGCCGCCGCTGGGTCCGCGCCGCCGCCCCTGCCACCGATGA
TGAGTGGAAGCCTACCGGCACCGTCCTCGTCACCGGCGGCACCGGCGGCGTCGGCGGCCAGATCGCCCGCTGGCTCGCCCG
CCGCGGCGCCCCTCATCTCCTCCTCGTCTCCCGCTCCGGCCCTGATGCCGATGGCGCCGGCGAGCTCGTCGCCGAGCTCGA
GGCCCTCGGCGCCCGCACCACCGTCGCCGCCTGCGATGTCACCGATCGCGAGTCCGTCCGCGAGCTCCTCGGCGGCATCGG

CGATGATGTCCCTCTCTCCGCCGTCTTCCATGCCGCCGCCACCCTCGATGATGGCACCGTCGATACCCTCACCGGCGAGCGC
ATCGAGCGCGCCTCCCGCGCCAAGGTCCTCGGCGCCCGCAACCTCCATGAGCTCACCCGCGAGCTCGATCTCACCGCCTTCG
TCCTCTTCTCCTCCTTCGCCTCCGCCTTCGGCGCCCCTGGCCTCGGCGGCTACGCCCCTGGCAACGCCTACCTCGATGGCCT
CGCCCAGCAGCGCCGCTCCGATGGCCTCCCTGCCACCGCCGTCGCCTGGGGCACCTGGGCCGGCTCCGGCATGGCCGAGG
GCCCTGTCGCCGATCGCTTCCGCCGCCATGGCGTCATCGAGATGCCTCCTGAGACCGCCTGCCGCGCCCTCCAGAACGCCCT
CGATCGCGCCGAGGTCTGCCCTATCGTCATCGATGTCCGCTGGGATCGCTTCCTCCTCGCCTACACCGCCCAGCGCCCTACC
CGCCTCTTCGATGAGATCGATGATGCCCGCCGCGCCGCCCCTCAGGCCGCCGCCGAGCCTCGCGTCGGCGCCCTCGCCTCC
CTCCCTGCCCCTGAGCGCGAGAAGGCCCTCTTCGAGCTCGTCCGCTCCCATGCCGCCGCCGTCCTCGGCCATGCCTCCGCCG
AGCGCGTCCCTGCCGATCAGGCCTTCGCCGAGCTCGGCGTCGATTCCCTCTCCGCCCTCGAGCTCCGCAACCGCCTCGGCGC
CGCCACCGGCGTCCGCCTCCCTACCACCACCGTCTTCGATCATCCTGATGTCCGCACCCTCGCCGCCCATCTCGCCGCCGAG
CTCGGCGGCGCCACCGGCGCCGAGCAGGCCGCCCCTGCCACCACCGCCCCTGTCGATGAGCCTATCGCCATCGTCGGCATG
GCCTGCCGCCTCCCTGGCGAGGTCGATTCCCCTGAGCGCCTCTGGGAGCTCATCACCTCCGGCCGCGATTCCGCCGCCGAG
GTCCCTGATGATCGCGGCTGGGTCCCTGATGAGCTCATGGCCTCCGATGCCGCCGGCACCCGCCGCGCCCATGGCAACTTCA
TGGCCGGCGCCGGCGATTTCGATGCCGCCTTCTTCGGCATCTCCCCTCGCGAGGCCCTCGCCATGGATCCTCAGCAGCGCCA
GGCCCTCGAGACCACCTGGGAGGCCCTCGAGTCCGCCGGCATCCCTCCTGAGACCCTCCGCGGCTCCGATACCGGCGTCTTC
GTCGGCATGTCCCATCAGGGCTACGCCACCGGCCGCCCTCGCCCTGAGGATGGCGTCGATGGCTACCTCCTCACCGGCAACA
CCGCCTCCGTCGCCTCCGGCCGCATCGCCTACGTCCTCGGCCTCGAGGGCCCTGCCCTCACCGTCGATACCGCCTGCTCCTC
CTCCCTCGTCGCCCTCCATACCGCCTGCGGCTCCCTCCGCGATGGCGATTGCGGCCTCGCCGTCGCCGGCGGCGTCTCCGTC
ATGGCCGGCCCTGAGGTCTTCACCGAGTTCTCCCGCCAGGGCGCCCTCTCCCCTGATGGCCGCTGCAAGCCTTTCTCCGATG
AGGCCGATGGCTTCGGCCTCGGCGAGGGCTCCGCCTTCGTCGTCCTCCAGCGCCTCTCCGATGCCCGCCGCGAGGGCCGCC
GCGTCCTCGGCGTCGTCGCCGGCTCCGCCGTCAACCAGGATGGCGCCTCCAACGGCCTCTCCGCCCCTTCCGGCGTCGCCC
AGCAGCGCGTCATCCGCCGCGCCTGGGCCCGCGCCGGCATCACCGGCGCCGATGTCGCCGTCGTCGAGGCCCATGGCACC
GGCACCCGCCTCGGCGATCCTGTCGAGGCCTCCGCCCTCCTCGCCACCTACGGCAAGTCCCGCGGCTCCTCCGGCCCTGTC
CTCCTCGGCTCCGTCAAGTCCAACATCGGCCATGCCCAGGCCGCCGCCGGCGTCGCCGGCGTCATCAAGGTCCTCCTCGGCC
TCGAGCGCGGCGTCGTCCCTCCTATGCTCTGCCGCGGCGAGCGCTCCGGCCTCATCGATTGGTCCTCCGGCGAGATCGAGCT
CGCCGATGGCGTCCGCGAGTGGTCCCCTGCCGCCGATGGCGTCCGCCGCGCCGGCGTCTCCGCCTTCGGCGTCTCCGGCAC
CAACGCCCATGTCATCATCGCCGAGCCTCCTGAGCCTGAGCCTGTCCCTCAGCCTCGCCGCATGCTCCCTGCCACCGGCGTC
GTCCCTGTCGTCCTCTCCGCCCGCACCGGCGCCGCCCTCCGCGCCCAGGCCGGCCGCCTCGCCGATCATCTCGCCGCCCAT
CCTGGCATCGCCCCTGCCGATGTCTCCTGGACCATGGCCCGCGCCCGCCAGCATTTCGAGGAGCGCGCCGCCGTCCTCGCC
GCCGATACCGCCGAGGCCGTCCATCGCCTCCGCGCCGTCGCCGATGGCGCCGTCGTCCCTGGCGTCGTCACCGGCTCCGCC
TCCGATGGCGGCTCCGTCTTCGTCTTCCCTGGCCAGGGCGCCCAGTGGGAGGGCATGGCCCGCGAGCTCCTCCCTGTCCCTG
TCTTCGCCGAGTCCATCGCCGAGTGCGATGCCGTCCTCTCCGAGGTCGCCGGCTTCTCCGTCTCCGAGGTCCTCGAGCCTCG
CCCTGATGCCCCTTCCCTCGAGCGCGTCGATGTCGTCCAGCCTGTCCTCTTCGCCGTCATGGTCTCCCTCGCCCGCCTCTGGC
GCGCCTGCGGCGCCGTCCCTTCCGCCGTCATCGGCCATTCCCAGGGCGAGATCGCCGCCGCCGTCGTCGCCGGCGCCCTCT
CCCTCGAGGATGGCATGCGCGTCGTCGCCCGCCGCTCCCGCGCCGTCCGCGCCGTCGCCGGCCGCGGCTCCATGCTCTCCG
TCCGCGGCGGCCGCTCCGATGTCGAGAAGCTCCTCGCCGATGATTCCTGGACCGGCCGCCTCGAGGTCGCCGCCGTCAACG
GCCCTGATGCCGTCGTCGTCGCCGGCGATGCCCAGGCCGCCCGCGAGTTCCTCGAGTACTGCGAGGGCGTCGGCATCCGCG
CCCGCGCCATCCCTGTCGATTACGCCTCCCATACCGCCCATGTCGAGCCTGTCCGCGATGAGCTCGTCCAGGCCCTCGCCGG
CATCACCCCTCGCCGCGCCGAGGTCCCTTTCTTCTCCACCCTCACCGGCGATTTCCTCGATGGCACCGAGCTCGATGCCGGCT
ACTGGTACCGCAACCTCCGCCATCCTGTCGAGTTCCATTCCGCCGTCCAGGCCCTCACCGATCAGGGCTACGCCACCTTCATC
GAGGTCTCCCCTCATCCTGTCCTCGCCTCCTCCGTCCAGGAGACCCTCGATGATGCCGAGTCCGATGCCGCCGTCCTCGGCA
CCCTCGAGCGCGATGCCGGCGATGCCGATCGCTTCCTCACCGCCCTCGCCGATGCCCATACCCGCGGCGTCGCCGTCGATTG
GGAGGCCGTCCTCGGCCGCGCCGGCCTCGTCGATCTCCCTGGCTACCCTTTCCAGGGCAAGCGCTTCTGGCTCCTCCCTGAT
CGCACCACCCCTCGCGATGAGCTCGATGGCTGGTTCTACCGCGTCGATTGGACCGAGGTCCCTCGCTCCGAGCCTGCCGCCC
TCCGCGGCCGCTGGCTCGTCGTCGTCCCTGAGGGCCATGAGGAGGATGGCTGGACCGTCGAGGTCCGCTCCGCCCTCGCCG
AGGCCGGCGCCGAGCCTGAGGTCACCCGCGGCGTCGGCGGCCTCGTCGGCGATTGCGCCGGCGTCGTCTCCCTCCTCGCCC
TCGAGGGCGATGGCGCCGTCCAGACCCTCGTCCTCGTCCGCGAGCTCGATGCCGAGGGCATCGATGCCCCTCTCTGGACCGT
CACCTTCGGCGCCGTCGATGCCGGCTCCCCTGTCGCCCGCCCTGATCAGGCCAAGCTCTGGGGCCTCGGCCAGGTCGCCTC
CCTCGAGCGCGGCCCTCGCTGGACCGGCCTCGTCGATCTCCCTCATATGCCTGATCCTGAGCTCCGCGGCCGCCTCACCGCC
GTCCTCGCCGGCTCCGAGGATCAGGTCGCCGTCCGCGCCGATGCCGTCCGCGCCCGCCGCCTCTCCCCTGCCCATGTCACC
GCCACCTCCGAGTACGCCGTCCCTGGCGGCACCATCCTCGTCACCGGCGGCACCGCCGGCCTCGGCGCCGAGGTCGCCCGC
TGGCTCGCCGGCCGCGGCGCCGAGCATCTCGCCCTCGTCTCCCGCCGCGGCCCTGATACCGAGGGCGTCGGCGATCTCACC
GCCGAGCTCACCCGCCTCGGCGCCCGCGTCTCCGTCCATGCCTGCGATGTCTCCTCCCGCGAGCCTGTCCGCGAGCTCGTCC
ATGGCCTCATCGAGCAGGGCGATGTCGTCCGCGGCGTCGTCCATGCCGCCGGCCTCCCTCAGCAGGTCGCCATCAACGATAT
GGATGAGGCCGCCTTCGATGAGGTCGTCGCCGCCAAGGCCGGCGGCGCCGTCCATCTCGATGAGCTCTGCTCCGATGCCGA
GCTCTTCCTCCTCTTCTCCTCCGGCGCCGGCGTCTGGGGCTCCGCCCGCCAGGGCGCCTACGCCGCCGGCAACGCCTTCCTC
GATGCCTTCGCCCGCCATCGCCGCGGCCGCGGCCTCCCTGCCACCTCCGTCGCCTGGGGCCTCTGGGCCGCCGGCGGCATG
ACCGGCGATGAGGAGGCCGTCTCCTTCCTCCGCGAGCGCGGCGTCCGCGCCATGCCTGTCCCTCGCGCCCTCGCCGCCCTC
GATCGCGTCCTCGCCTCCGGCGAGACCGCCGTCGTCGTCACCGATGTCGATTGGCCTGCCTTCGCCGAGTCCTACACCGCCG
CCCGCCCTCGCCCTCTCCTCGATCGCATCGTCACCACCGCCCCTTCCGAGCGCGCCGGCGAGCCTGAGACCGAGTCCCTCCG
CGATCGCCTCGCCGGCCTCCCTCGCGCCGAGCGCACCGCCGAGCTCGTCCGCCTCGTCCGCACCTCCACCGCCACCGTCCT
CGGCCATGATGATCCTAAGGCCGTCCGCGCCACCACCCCTTTCAAGGAGCTCGGCTTCGATTCCCTCACCGCCGTCGGCCTC
CGCAACCAGCTCCAGCAGGCCACCGGCCTCGCCCTCCCTGCCACCCTCGTCTTCGAGCATCCTACCGTCCGCCGCCTCGCCG
ATCATATCGGCCAGCAGCTCGATTCCGGCACCCCTGCCCGCGAGGCCTCCTCCGCCCTCCGCGATGGCTACCGCCAGGCCGG
CGTCTCCGGCCGCGTCCGCTCCTACCTCGATCTCCTCGCCGGCCTCTCCGATTTCCGCGAGCATTTCGATGGCTCCGATGGCT
TCTCCCTCGATCTCGTCGATATGGCCGATGGCCCTGGCGAGGTCACCGTCATCTGCTGCGCCGGCACCGCCGCCATCTCCGG
CCCTCATGAGTTCACCCGCCTCGCCGGCGCCCTCCGCGGCATCGCCCCTGTCCGCGCCGTCCCTCAGCCTGGCTACGAGGA
GGGCGAGCCTCTCCCTTCCTCCATGGCCGCCGTCGCCGCCGTCCAGGCCGATGCCGTCATCCGCACCCAGGGCGATAAGCCT
TTCGTCGTCGCCGGCCATTCCGCCGGCGCCCTCATGGCCTACGCCCTCGCCACCGAGCTCCTCGATCGCGGCCATCCTCCTC
GCGGCGTCGTCCTCATCGATGTCTACCCTCCTGGCCATCAGGATGCCATGAACGCCTGGCTCGAGGAGCTCACCGCCACCCT

CTTCGATCGCGAGACCGTCCGCATGGATGATACCCGCCTCACCGCCCTCGGCGCCTACGATCGCCTCACCGGCCAGTGGCGC
CCTCGCGAGACCGGCCTCCCTACCCTCCTCGTCTCCGCCGGCGAGCCTATGGGCCCTTGGCCTGATGATTCCTGGAAGCCTA
CCTGGCCTTTCGAGCATGATACCGTCGCCGTCCCTGGCGATCATTTCACCATGGTCCAGGAGCATGCCGATGCCATCGCCCGC
CATATCGATGCCTGGCTCGGCGGCGGCAACTCCTGA

Codon-optimized PccA:

ATGCGCAAGGTCCTCATCGCCAACCGCGGCGAGATCGCCGTCCGCGTCGCCCGCGCCTGCCGCGATGCCGGCATCGCCTCC
GTCGCCGTCTACGCCGATCCTGATCGCGATGCCCTCCATGTCCGCGCCGCCGATGAGGCCTTCGCCCTCGGCGGCGATACCC
CTGCCACCTCCTACCTCGATATCGCCAAGGTCCTCAAGGCCGCCCGCGAGTCCGGCGCCGATGCCATCCATCCTGGCTACGG
CTTCCTCTCCGAGAACGCCGATTTCGCCCAGGCCGTCCTCGATGCCGGCCTCATCTGGATCGGCCCTCCTCCTCATGCCATCC
GCGATCGCGGCGAGAAGGTCGCCGCCCGCCATATCGCCCAGCGCGCCGGCGCCCCTCTCGTCGCCGGCACCCCTGATCCTG
TCTCCGGCGCCGATGAGGTCGTCGCCTTCGCCAAGGAGCATGGCCTCCCTATCGCCATCAAGGCCGCCTTCGGCGGCGGCG
GCCGCGGCCTCAAGGTCGCCCGCACCCTCGAGGAGGTCCCTGAGCTCTACGATTCCGCCGTCCGCGAGGCCGTCGCCGCCT
TCGGCCGCGGCGAGTGCTTCGTCGAGCGCTACCTCGATAAGCCTCGCCATGTCGAGACCCAGTGCCTCGCCGATACCCATGG
CAACGTCGTCGTCGTCTCCACCCGCGATTGCTCCCTCCAGCGCCGCCATCAGAAGCTCGTCGAGGAGGCCCCTGCCCCTTTC
CTCTCCGAGGCCCAGACCGAGCAGCTCTACTCCTCCTCCAAGGCCATCCTCAAGGAGGCCGGCTACGTCGGCGCCGGCACCG
TCGAGTTCCTCGTCGGCATGGATGGCACCATCTCCTTCCTCGAGGTCAACACCCGCCTCCAGGTCGAGCATCCTGTCACCGAG
GAGGTCGCCGGCATCGATCTCGTCCGCGAGATGTTCCGCATCGCCGATGGCGAGGAGCTCGGCTACGATGATCCTGCCCTCC
GCGGCCATTCCTTCGAGTTCCGCATCAACGGCGATCATCCTGGCCGCGGCTTCCTCCCTGCCCCTGGCACCGTCACCCTCTTC
GATGCCCCTACCGGCCCTGGCGTCCGCCTCGATGCCGGCGTCGAGTCCGGCTCCGTCATCGGCCCTGCCTGGGATTCCCTCC
TCGCCAAGCTCATCGTCACCGGCCGCACCCGCGCCGAGGCCCTCCAGCGCGCCGCCCGCGCCCTCGATGAGTTCACCGTCG
AGGGCATGGCCACCGCCATCCCTTTCCATCGCACCGTCGTCCGCGATCCTGCCTTCGCCCCTGAGCTCACCGGCTCCACCGA
TCCTTTCACCGTCCATACCCGCTGGATCGAGACCGAGTTCGTCAACGAGATCAAGCCTTTCACCACCCCTGCCGATACCGAGA
CCGATGAGGAGTCCGGCCGCGAGACCGTCGTCGTCGAGGTCGGCGGCAAGCGCCTCGAGGTCTCCCTCCCTTCCTCCCTCG
GCATGTCCCTCGCCCGCACCGGCCTCGCCGCCGGCGCCCGCCCTAAGCGCCGCGCCGCCAAGAAGTCCGGCCCTGCCGCCT
CCGGCGATACCCTCGCCTCCCCTATGCAGGGCACCATCGTCAAGATCGCCGTCGAGGAGGGCCAGGAGGTCCAGGAGGGCG
ATCTCATCGTCGTCCTCGAGGCCATGAAGATGGAGCAGCCTCTCAACGCCCATCGCTCCGGCACCATCAAGGGCCTCACCGC
CGAGGTCGGCGCCTCCCTCACCTCCGGCGCCGCCATCTGCGAGATCAAGGATTGA

Codon-optimized PccB:

ATGTCCGAGCCTGAGGAGCAGCAGCCTGATATCCATACCACCGCCGGCAAGCTCGCCGATCTCCGCCGCCGCATCGAGGAGG
CCACCCATGCCGGCTCCGCCCGCGCCGTCGAGAAGCAGCATGCCAAGGGCAAGCTCACCGCCCGCGAGCGCATCGATCTCC
TCCTCGATGAGGGCTCCTTCGTCGAGCTCGATGAGTTCGCCCGCCATCGCTCCACCAACTTCGGCCTCGATGCCAACCGCCCT
TACGGCGATGGCGTCGTCACCGGCTACGGCACCGTCGATGGCCGCCCTGTCGCCGTCTTCTCCCAGGATTTCACCGTCTTCG
GCGGCGCCCTCGGCGAGGTCTACGGCCAGAAGATCGTCAAGGTCATGGATTTCGCCCTCAAGACCGGCTGCCCTGTCGTCGG
CATCAACGATTCCGGCGGCGCCCGCATCCAGGAGGGCGTCGCCTCCCTCGGCGCCTACGGCGAGATCTTCCGCCGCAACAC
CCATGCCTCCGGCGTCATCCCTCAGATCTCCCTCGTCGTCGGCCCTTGCGCCGGCGGCGCCGTCTACTCCCCTGCCATCACC
GATTTCACCGTCATGGTCGATCAGACCTCCCATATGTTCATCACCGGCCCTGATGTCATCAAGACCGTCACCGGCGAGGATGTC
GGCTTCGAGGAGCTCGGCGGCGCCCGCACCCATAACTCCACCTCCGGCGTCGCCCATCATATGGCCGGCGATGAGAAGGAT
GCCGTCGAGTACGTCAAGCAGCTCCTCTCCTACCTCCCTTCCAACAACCTCTCCGAGCCTCCTGCCTTCCCTGAGGAGGCCGA
TCTCGCCGTCACCGATGAGGATGCCGAGCTCGATACCATCGTCCCTGATTCCGCCAACCAGCCTTACGATATGCATTCCGTCAT
CGAGCATGTCCTCGATGATGCCGAGTTCTTCGAGACCCAGCCTCTCTTCGCCCCTAACATCCTCACCGGCTTCGGCCGCGTCG
AGGGCCGCCCTGTCGGCATCGTCGCCAACCAGCCTATGCAGTTCGCCGGCTGCCTCGATATCACCGCCTCCGAGAAGGCCGC
CCGCTTCGTCCGCACCTGCGATGCCTTCAACGTCCCTGTCCTCACCTTCGTCGATGTCCCTGGCTTCCTCCCTGGCGTCGATC
AGGAGCATGATGGCATCATCCGCCGCGGCGCCAAGCTCATCTTCGCCTACGCCGAGGCCACCGTCCCTCTCATCACCGTCATC
ACCCGCAAGGCCTTCGGCGGCGCCTACGATGTCATGGGCTCCAAGCATCTCGGCGCCGATCTCAACCTCGCCTGGCCTACCG
CCCAGATCGCCGTCATGGGCGCCCAGGGCGCCGTCAACATCCTCCATCGCCGCACCATCGCCGATGCCGGCGATGATGCCG
AGGCCACCCGCGCCCGCCTCATCCAGGAGTACGAGGATGCCCTCCTCAACCCTTACACCGCCGCCGAGCGCGGCTACGTCGA
TGCCGTCATCATGCCTTCCGATACCCGCCGCCATATCGTCCGCGGCCTCCGCCAGCTCCGCACCAAGCGCGAGTCCCTCCCT
CCTAAGAAGCATGGCAACATCCCTCTCTAA

Codon-optimized PccE:

ATGACCATCAAGGTCGTCCGCGGCAACCCTACCCCTGAGGAGCTCGCCGCCGCCCTCACCGTCGTCCGCGCCCGCGCCGTCA
CCGCCGCCGCCGAGCCTTCCACCACCGATCGCCCTCATGATGCCTGGTCCGATCCTTCCCGCATCGCCACCCATCATATGCCT
CATCCTGGCCCTACCGCCTGGGGCCGCACCTACTGGCCTACCTGA

4. DEBS1-TE Expression Cassette Construction

The vector pTYGS·*arg* was firstly chosen for *pcc* plasmid construction. All *pcc* gene fragments flanked with overlaps (by *ca* 30 bp) were synthesized commercially. Using an *Asc*I-digested vector pTYGS·*arg* and recovered *pccABE* gene fragments after double restriction enzymatic digestions (*pccA*-*EcoR*I/*BamH*I, *pccB*-*Xba*I/*BamH*I, *pccE*-*BamH*I/*Xba*I), the yeast recombination was performed as previously published protocols.[1]

Yeast colonies were obtained and then collected together to extract plasmids. Subsequently, using standard heat-shock protocols, the extracted plasmid mixture was introduced into *E. coli ccdB* survival 2 $T1^R$, which is resistant against the CcdB killer protein. A large number of *E. coli* colonies were generated, and then 5 random colonies were identified by colony PCR and restriction enzyme digestion. Lastly, the expression plasmid was successfully constructed (Scheme S4.1). Three subunit genes were inserted downstream of promoters P_{adh}, P_{gpdA}, and P_{eno}, respectively.

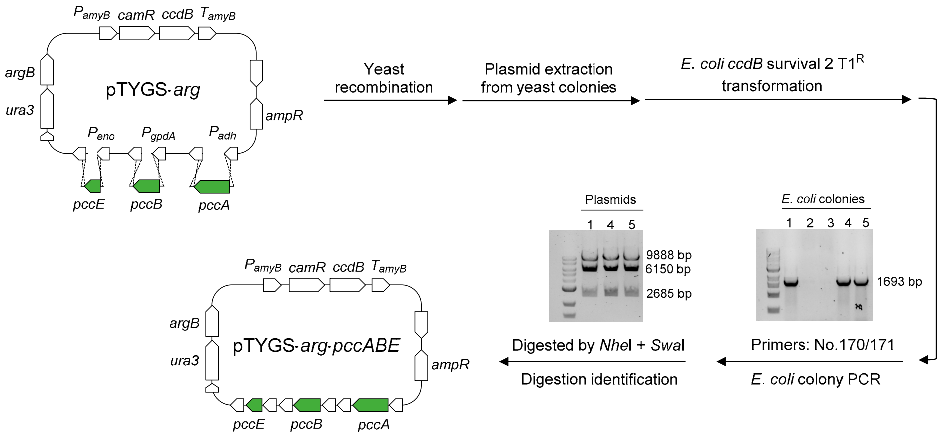

Scheme S4.1 Construction of the plasmid pTYGS·*arg*·*pccABE*. For *E. coli* colony PCR, the *pccB* was chosen to perform PCR identification. Primers No.170/171 are located in the P_{gpdA} and T_{gpdA}, respectively. For plasmid digestion identification, restriction enzymes *Nhe*I+*Swa*I were chosen. All of DNA bands above were shown as positive.

The vector pEYA was chosen for DEBS1-TE plasmid construction. For the synthetic convenience, the entire DEBS1-TE sequence (~11 kb) was designed as four fragments (~2.8 kb each). Each fragment was designed to overlap with adjacent fragments or the expression vector at both ends (by *ca* 30 bp) for the subsequent yeast recombination. The initiation end of the first fragment and

the termination end of the fourth fragment were respectively attached with homologous fragments, which aim for yeast recombination with the linearized pEYA vector. To avoid the inaccessibility of PCR product due to high GC content, restriction enzyme digestion was required to obtain all desired fragments. Therefore, two different unique restriction enzymes were adhered to ends of each fragment (*debs1te·f1*-*Sca*I/*BamH*I; *debs1te·f2*-*Sma*I/*Hand*III; *debs1te·f3*-*EcoR*V/*Xba*I; *debs1te·f4*-*Dra*I/*EcoR*I).

The four DEBS1-TE fragments were synthesized commercially in four separate pUC57 vectors. All desired DEBS1-TE fragments and the linearized pEYA vector were prepared by restriction-enzyme digestion and then introduced into competent yeast for homologous recombination (Scheme S2).[1] Two negative controls were set up. After two days of incubation, yeast colonies were produced only on the experimental sample. Then, plasmids were extracted from yeast colonies and immediately introduced into *E. coli* Top10 competent. *E. coli* colonies were screened by PCR identification. In the resulting positive *E. coli* colonies, plasmids were extracted and then identified by restriction enzyme digestion (Scheme S4.2). The empty vector pEYA was used as the control. The extract plasmids were digested by the restriction enzyme *Sca*I and formed two bands (*i.e.* 9182 bp and 7466 bp) which match the predicted sizes. Ultimately, the obtained plasmid was confirmed to be pEYA·*debs1te* by gene sequencing.

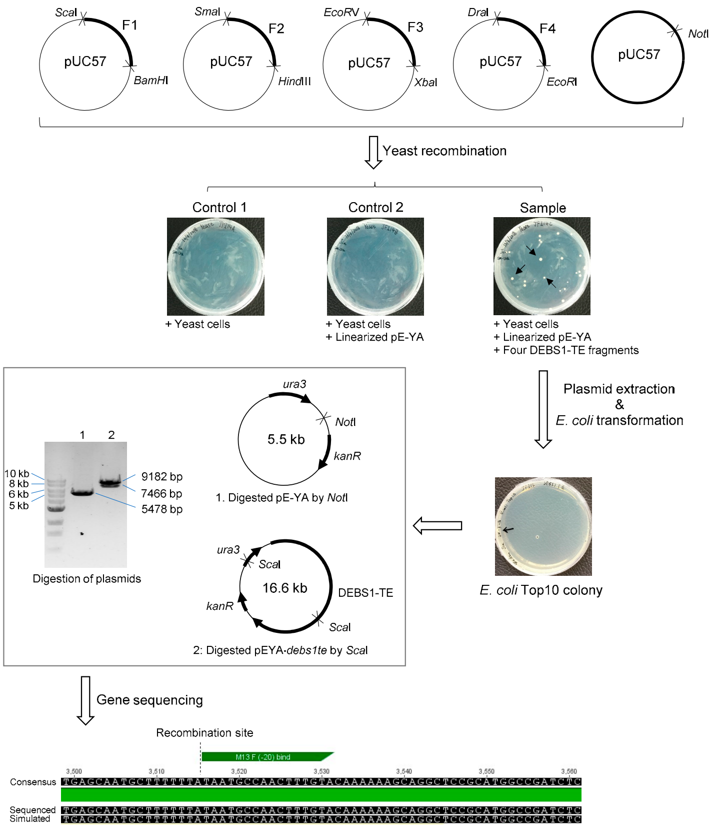

Scheme S4.2 Yeast recombination, colony screening, and identification of the plasmid pEYA·*debs1te* by restriction enzymatic digestion and gene sequencing.

To construct the final plasmid for DEBS1-TE expression, LR recombination was carried out using pEYA·*debs1te* as the entry clone and pTYGS·*arg·pccABE* as the destination vector.[2] It led to the formation of the plasmid pTYGS·*arg·debs1te-pccABE* harboring *pccABE* genes and DEBS1-TE simultaneously, of which DEBS1-TE locates downstream of the promotor P_{amyB}.

5. Gene Expression Identification of DEBS1-TE and PCC

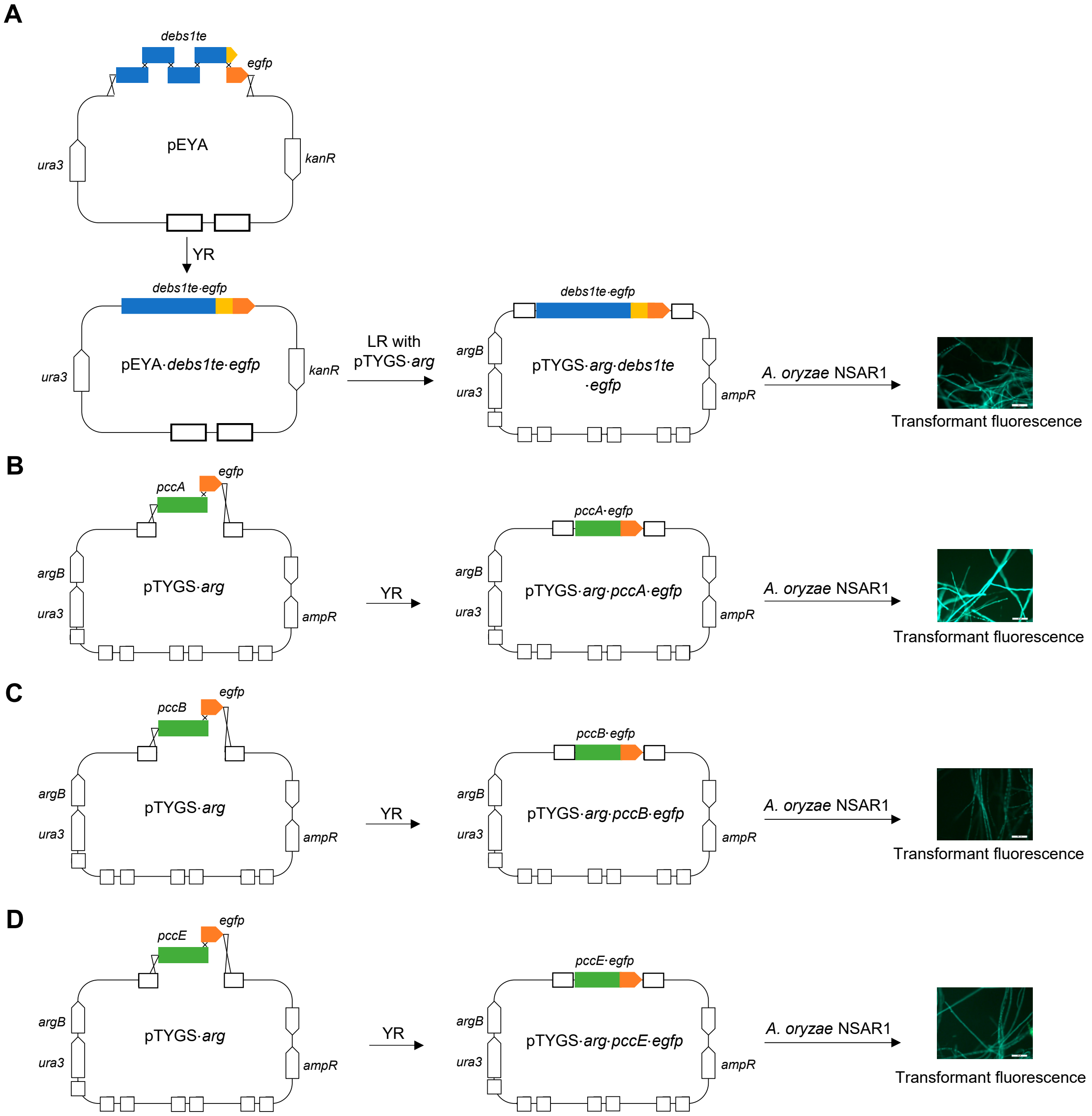

Scheme S5.1 Green fluorescent imaging of transformants with biosynthetic genes fused to *egfp*: **A**, *egfp* was fused with DEBS1-TE; **B**, *egfp* was fused with *pccA*; **C**, *egfp* was fused with *pccB*; **D**, *egfp* was fused with *pccE*. Abbreviations: YR, yeast recombination; LR, Gateway LR recombination.

6. Fungal and Bacterial PPTase Alignment

```
S.erythraea-SePptII     --------------------------------mierv1pegatwveafddpaeat1fpee     28
B.subtilis-Sfp          -----------mkiygiymd-rp1s-qeenerfmtfispekrekcrrfyh--------ke     39
A.oryzae-PPTase         --mqppqdessncmvrwyidtrd1tatttslp11et1qppdqesakrfyh--------1k     50
A.nidulans-NpgA         mvqdtssastspi1trwyidtrp1tastaa1p11et1qpadqisvqkyyh--------1k     52
                                                        ::  : *      . : .         :

S.erythraea-SePptII     eaaiaravdkrrrefttvrhcarramae1gvppap11pgergapqw--------------     74
B.subtilis-Sfp          dahrt11gdv1vrsv-isrqyq1-dksdirfst---q--eygkpci--------------     78
A.oryzae-PPTase         dkhms1asn11ky1f-ihrtcri-pwnqitisr---tpaphhrpyfnaagfiqtaa--td     103
A.nidulans-NpgA         dkhms1asn11ky1f-vhrncri-pwssivisr---tpdphrrpcyippsgsqedsfkdg     107
                        :   :   :     .   *        .: .            *

S.erythraea-SePptII     --------pagvvgsmthcagy-------------------raavvgpagtvvtigidae     107
B.subtilis-Sfp          pd1pdahfnishsgrwvigafd-----------------------------sqpigidie     109
A.oryzae-PPTase         kpipniefnvshqas1va1agti1ppssnndsiaptnvitnpnptstpassipqvgidit     163
A.nidulans-NpgA         ytginvefnvshqasmvaiagtaftpnsggds-----------------k1kpevgidit     150
                                  .  .  .  *                                  :***

S.erythraea-SePptII     phep1pggv1davs1pe---------------erar1re1-------stqdgkvhwdrv1     145
B.subtilis-Sfp          ktkp--------is1-------eiakrffskteysd1--------1akdk----deqtdy     142
A.oryzae-PPTase         cvnerrntpetrqa1ed1hgvp---------------------shigndedg1veygfr1     202
A.nidulans-NpgA         cvnerqgrngeers1es1rqyidifsevfstaemanirr1dgvssss1sadr1vdygyr1     210
                          :          :*                                       .

S.erythraea-SePptII     fsckesvykawfp1tgaw1dfeqad1tfdaaggtfhar11kpgg-qaegrp1teftgrw1     204
B.subtilis-Sfp          fy-------h1wsmkesfikqegkg1s1p1dsfsvr1hqdgqvsie1pdshspcyiktye     195
A.oryzae-PPTase         fy-------tywa1keayikmtgea11apw1re1vftnv1apep---agrh1htwgepyt     252
A.nidulans-NpgA         fy-------tywa1keayikmtgea11apw1re1efsnvvapaavaesgdsagdfgepyt     263
                        *          : :. :::.     *           .          .     :   :

S.erythraea-SePptII     aadgfvvsai---------vr1rer-----------------------------------     220
B.subtilis-Sfp          vdpgykmavcaahpdfpedi--tmvsye--e11---------------------------     224
A.oryzae-PPTase         ---gvktw1y---gkevedvr1evvafendy1iataargggigwrsegd---------ga     297
A.nidulans-NpgA         ---gvrtt1y---kn1vedvrievaa1ggdy1fataargggigassrpgggpdgsgirsq     317
                           *               :

S.erythraea-SePptII     ---------------------------          220
B.subtilis-Sfp          ---------------------------          224
A.oryzae-PPTase         dpwqr1ekidiekdvrpcatgvcqc1k          324
A.nidulans-NpgA         dpwrpfkk1dierdiqpcatgvcnc1s          344
```

Figure S6.1 Sequence alignment of different sources of PPTases (the conserved motifs are highlighted in grey): *S. erythraea* SePptII accession number AAR92400, *B. subtilis* Sfp accession number CAA44858, *A. oryzae* PPTase accession number XP_023093666 and *A. nidulans* NpgA accession number AAF12814.

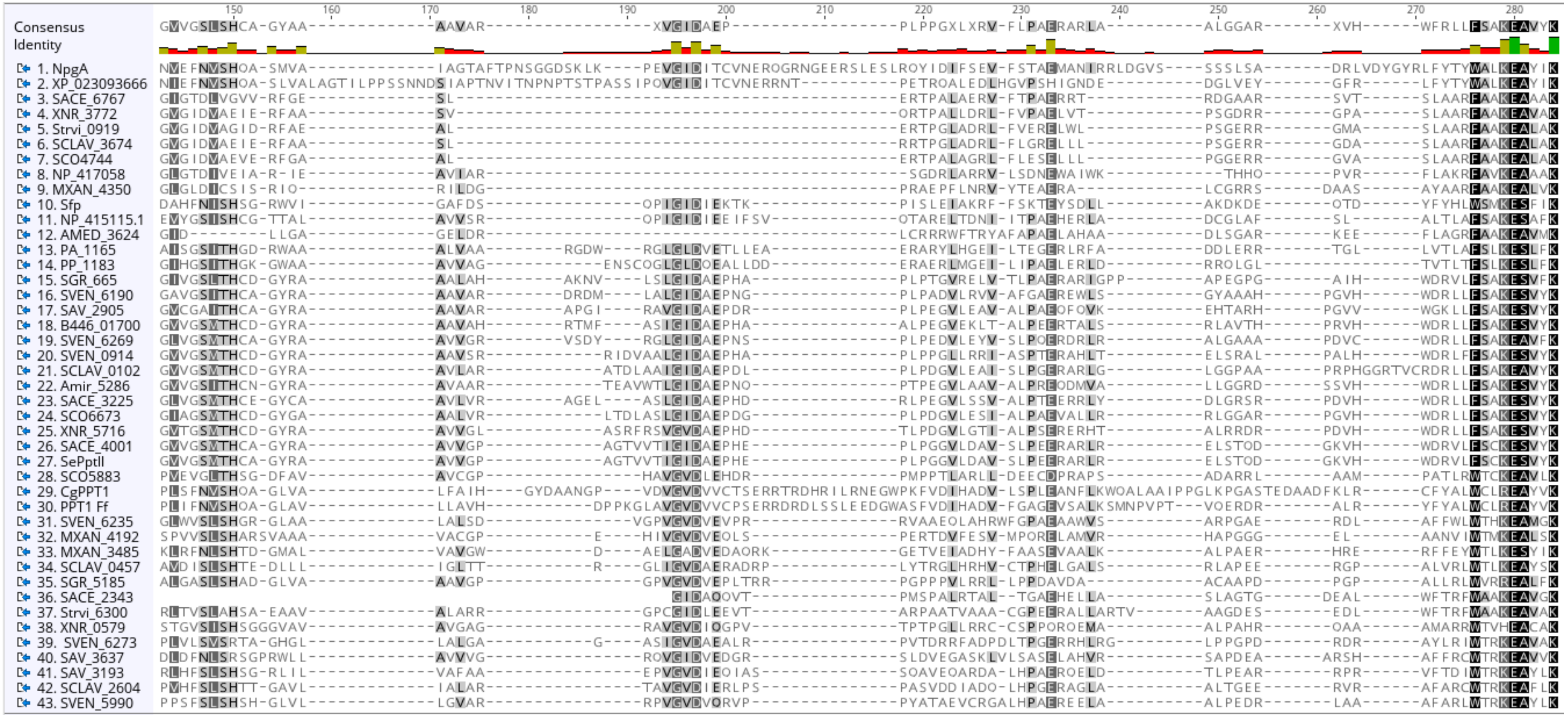

Figure 6.2: Sequence alignment analysis of a wider range of PPTases (adapted from reference 38) displaying the variation in the FxxKESxxK and WxxKEAAxxK subtypes.

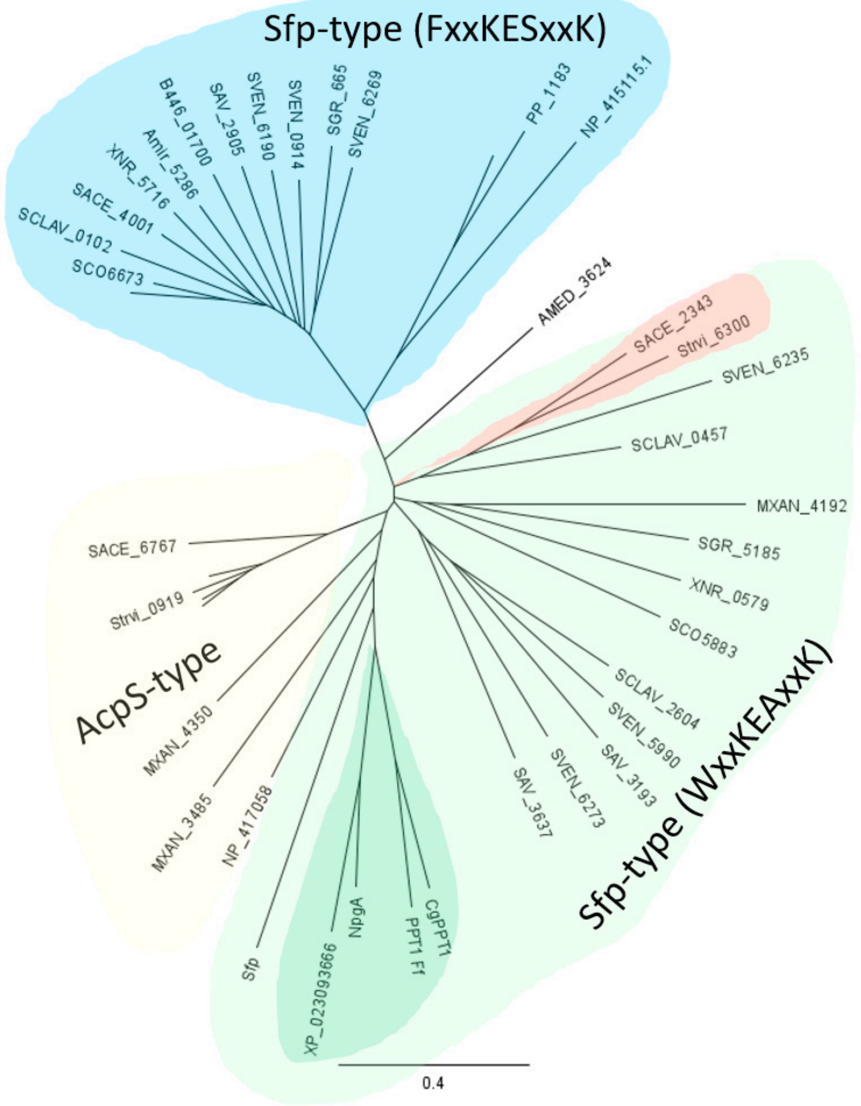

Figure 6.3: Phylogenetic analysis of bacterial and fungal PPT. Colour code indicates the different types: yellow = AcpS-type; blue = Sfp type 1 (FxxKESxxK); green = Sfp type 2 (WxxKEAxxK), red = PKS / FAS integrated PPT. Fungal PPTs form a sub-clade within the WxxKEAxxK Sfp family shaded in dark green.

Codon-optimized Sfp:

ATGAAGATTTACGGAATTTATATGGACCGCCCGCTTTCCCAGGAAGAGAATGAACGGTTCATGTCTTTCATATCGCCTGAAAAAC
AGGAGAAATGCCGGAGATTTTATCATAAAGAAGATGCTCACCGCACCCTGCTAGGAGATGTGCTCGTTCGCTCGGTTATTAGCA
GGCAGTATCAGCTGGACAAAGCTGACATCCGCTTCAGCGCGCAGGAATACGGGAAGCCTTGCATCCCTGATCTTCCTGACGCC
CATTTCAACATTTCTCACTCCGGCCGCTGGGTCATTTGTGCGTTTGATTCACACCCGATCGGCATCGATATTGAAAAAATGAAAC
CGATCAGCCTTGAGATCGCCAAACGTTTCTTTTCAAAAACAGAGTACAGCGACCTTTTAGCAAAAAACAAGGACGAGCAGACAG
ACTATTTTTATCATCTATGGTCAATGAAAGAAAGCTTTATCAAACAGGAAGGCAAAGGCTTATCACTTCCGCTAGATTCCTTTTCA
GTGCGCCTGCATCAGGACGGACAAGTATCCATTGAGCTTCCGGACAGCCATACACCATGCTATATCAAAACCTATGAGGTGGAT
CCCGGCTACAAAATGGCCGTATGCGCCGCGCACCCTGATTTCCCCGAGGATATCACAATGCTCTCGTACGAAGCGCTTTTATAA

Codon-optimized SePptII:

ATGATAGAGAGGGTTCTCCCCGAAGGTGCTACCTGGGTAGAAGCATTCGATGATCCAGCAGAGGCTACATTATTCCCCGAAGA
GGAAGCAGCGATTGCCCGCGCTGTTGATAAAAGACGGAGAGAATTTACTACTGTTAGGCATTGTGCGCGACGAGCGATGGCCG
AACTCGGAGTTCCCCCTGCCCCTCTACTGCCAGGCGAGCGAGGAGCTCCACAGTGGCCGGCTGGCGTAGTTGGCTCAATGAC
TCATTGTGCTGGATACCGGGCAGCGGTCGTAGGACCTGCTGGAACTGTCGTAACGATAGGAATCGACGCGGAACCGCATGAA
CCATTACCTGGCGGTGTACTAGATGCTGTTTCTTTGCCAGAGGAAAGAGCGCGCCTACGGGAACTATCAACCCAGGATGGCAA
AGTTCATTGGGATAGAGTGTTATTCTCTTGTAAGGAAAGTGTGTATAAGGCCTGGTTCCCGCTGACCGGGGCGTGGCTGGACTT
TGAACAAGCGGATCTCACGTTCGATGCTGCTGGCGGGACCTTCCATGCACGGTTACTCAAACCTGGTGGCCAGGCGGAAGGT
CGACCGCTGACAGAATTTACTGGTCGTTGGTTAGCCGCAGACGGATTTGTGGTGTCTGCCATCGTCCGACTTAGAGAACGGTG
A

7. Synthesis of TKL

General

All reactions were performed in oven-dried glassware and under a nitrogen atmosphere. For air or moisture-sensitive reactions the glassware was evacuated and heated out by a heating gun additionally (Schlenk conditions). A magnetic stirrer was used for every reaction. Reactions requiring cooling to -78 °C, a mixture of acetone and dry ice were used. With an ice bath, reactions were cooled to 0 °C. Reactions were heated with silicon oil as a heat bath. The temperature was adjusted by a contact thermometer. Removal of solvents was performed under reduced pressure using a rotary evaporator with a 40 °C water bath temperature unless otherwise stated. All reagents and solvents were used as received from commercial suppliers abcr GmbH, Fisher Chemicals, or Sigma Aldrich unless indicated otherwise. Diisopropylamine was distilled over calcium hydride and stored under an inert nitrogen atmosphere.

Thin-layer chromatography was performed on aluminum-backed silica gel plates F254 of Merck (median pore size 60 Å, layer thickness 0.2 mm, particle size: 5-17 µm) and detected with Cerium (IV) or $KMnO_4$ dip-reagents and if necessary subsequent heating with a heating gun.

Column flash chromatography was used for purification and performed using the silica gel of Merck with average particle diameter 50 µM (range 40-63 µM, pore diameter 53 Å), and eluents are given in brackets. Column diameter and filling level were adapted to the purification according to the reaction mixture or reaction scale.

LCMS data of the reaction mixture or organic extracts were obtained either by a Waters mass-directed auto purification system (Waters, Milford, Massachusetts, USA) including a Waters 2767 autosampler and a Waters 2545 binary gradient module system, or by a Waters 2795 alliance HT separations module. Both systems are equipped with a Phenomenex Kinetex column (2.6 µm, C_{18}, 100 Å, 4.6 × 100 mm; Phenomenex, Torrance, California, USA) and a Phenomenex Security Guard precolumn (Luna, C_5, 300 Å). Detection methods are a diode array detector from 210-600 nm (DAD; Waters 2998 or Waters 996), an electron light scattering detector (ELSD; Wat*ers 2424) and an electrospray ionization mass detector in the range of 100-1000* m/z (Waters SQD-2 or Waters Micromass ZQ). Gradient was run over 15 min starting at 10 % acetonitrile/ 90 % HPLC grade water and increasing to 90 % acetonitrile/ 10 % HPLC grade water. Acetonitrile was mixed

with 0.05 % formic acid and water with 0.045 % formic acid. The flow rate was 1 mL/min and 20 μL of the sample were injected. Data were displayed using the software MassLynx.

The preparative LCMS was used for the purification of compounds from the organic extracts by a Waters mass–directed auto purification system. It is equipped with a Waters 2767 autosampler, a Waters 2545 binary gradient module system, a Phenomenex Kinetex Axia column (5 μm, C_{18}, 100 Å, 21.2 × 250 mm), and a Phenomenex Security Guard precolumn (Luna, C_5, 300 Å). Gradient was run over 15 min starting at 10 % acetonitrile/ 90 % HPLC grade water (+ 0.05 % formic acid) and increasing to 90 % acetonitrile/ 10 % HPLC grade water (+ 0.05 % formic acid). The flow rate was 20 mL/min and the post-column flow was split (100:1). The minority flow was made up with HPLC grade acetonitrile + 0.05 % formic acid to a flow rate of 1 mL/min for simultaneous analysis by a diode array detector (DAD, Waters 2998) in the range of 200 to 600 nm, an electron light scattering detector (ELSD; Waters 2424) and a Waters SQD-2 electrospray ionization mass detector in the range of 100 and 1000 m/z. Selected peaks were collected and the solvent was evaporated overnight using a freeze dryer.

NMR spectra were recorded on a Bruker Ascend 400 MHz, Bruker Ultrashield 400MHz, Bruker Ultrashield 500 MHz, or Bruker Ascend 600 MHz spectrometer at 298 K unless otherwise stated and calibrated by using the residual peak of the solvent as the internal standard ($CDCl_3$: δ_H = 7.26 ppm; δ_C = 77.2 ppm; CD_3OD: δ_H = 3.31 ppm; δ_C = 49.0 ppm). Two-dimensional proton-carbon correlation techniques (HSQC and HMBC) were used to determine the structure or supplement and complete the ^{13}C-NMR spectra where signals were otherwise broadened or weak. All spectra were evaluated with MestReNova of Mestrelab Research S. L.

S-4-benzyl-3-(2S,3R-3-hydroxy-2-methylpentanoyl)oxazolidin-2-one[3]

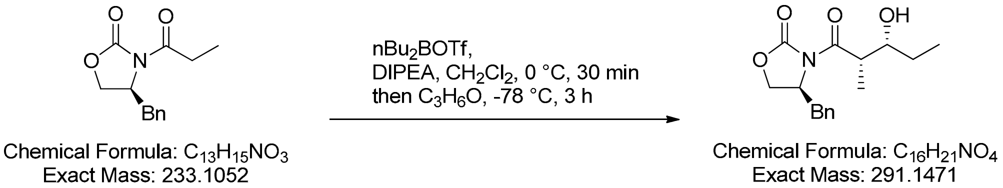

S-4-benzyl-3-propionyloxazolidin-2-one (1.0 g, 4.18 mmol, 1.0 equiv.) was dissolved in anhydrous CH_2Cl_2 (25 mL) and cooled to 0 °C. To the stirred solution was added *n*Bu_2BOTf (1.0 M in CH_2Cl_2, 4.7 mL, 4.7 mmol, 1.1 equiv.) dropwise over 15 min. After addition, DIPEA (1.2 mL, 6 mmol, 1.4 equiv.) was added, and stirring continued for an additional 15 min. Then the solution was cooled to -78 °C and propionaldehyde (0.68 mL, 9.42 mmol, 2.2 equiv.) was added dropwise. After addition, the solution was stirred for 3h at -78°C, before the reaction was allowed to warm to 0 °C. The reaction was quenched with potassium phosphate buffer (pH 7.1, 15 mL), MeOH (8 mL), and MeOH/30 %-H_2O_2 (25 mL, 2:1). After stirring 1 h at 0 °C the aqueous layer was extracted with CH_2Cl_2 (3 × 50 mL). The combined organic layers were washed with saturated $NaHCO_3$- and NaCl-Solution, dried over $MgSO_4$, filtered, and concentrated under reduced pressure. The crude aldol product was used in the next step without further purification.

S-4-benzyl-3-(2S,3R-3-((tert-butyldimethylsilyl)oxy)-2-methylpentanoyl)oxazolidin-2-one[3]

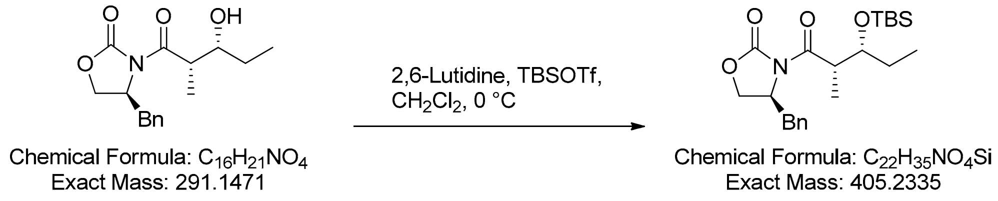

S-4-benzyl-3-(2*S*,3*R*-3-hydroxy-2-methylpentanoyl)oxazolidin-2-one (1.26 g, 4.32 mmol, 1.0 equiv.) was dissolved in anhydrous CH_2Cl_2 (20 mL) and cooled to 0 °C. Then 2,6-Lutidine (0.75 mL, 6.48 mmol, 1.5 equiv.) and TBSOTf (1.5 mL, 6.48 mmol, 1.5 equiv.) were added dropwise. After stirring for 1 h, the solution was allowed to warm to room temperature and stirred for an additional 30 min. After completion, the reaction was quenched by the addition of saturated NH_4Cl-solution (15 mL). The aqueous phase was extracted with CH_2Cl_2 (3 × 20 mL), dried over $MgSO_4$, filtered, and concentrated under reduced pressure. The crude product was dissolved in CH_2Cl_2 and purified by flash chromatography on silica gel (PE:EtOAc = 4:1). The TBS-protected product was obtained as a colorless solid (1.6 g, 3,94 mmol, 94 % over two steps). **R_f** = 0.78 (PE:EtOAc = 4:1); **^{1}H NMR** (400 MHz, $CDCl_3$) δ = 7.35-7.27 (m, 3H, Bn), 7.23-7.21 (m, 2H, Bn), 4.60 (ddt, J = 9.5, 6.5, 3.2 Hz, 1H, NC*H*Bn), 4.19-4.16 (m, 2H, OC*H*$_2$CHBn), 3.96 (q, J = 5.6 Hz, 1H, C*H*(OTBS)CH_2), 3.91-3.85 (m, 1H, C(O)C*H*CH_3), 3.30 (dd, J = 13.3, 3.1 Hz, 1H, BnC*H*$_2$), 2.77 (dd, J = 13.3, 9.7 Hz, 1H, BnC*H*$_2$), 1.60-1.52 (m, 2H, CH_3C*H*$_2$CH), 1.21 (d, J = 6.8 Hz, 3H, C*H*$_3$CHC(O)), 0.89 (m, 9H, OSiC(C*H*$_3$)$_3$), 0.86 (s, 3H, C*H*$_3$$CH_2$), 0.04 (s, 3H, OSiC*H*$_3$C($CH_3$)$_3$), 0.01 (s, 3H, OSiC*H*$_3$C($CH_3$)$_3$).

2*R*,3*R*-3-((tert-butyldimethylsilyl)oxy)-2-methylpentan-1-ol[4]

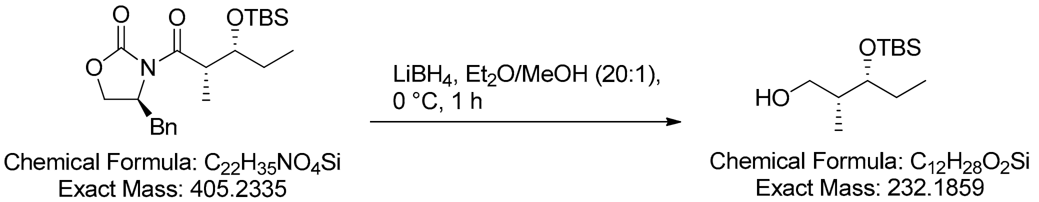

S-4-benzyl-3-(2*S*,3*R*-3-((tert-butyldimethylsilyl)oxy)-2-methylpentanoyl)oxazolidin-2-one (1.0 g, 2.46 mmol, 1.0 equiv.) was dissolved in Et_2O:MeOH (25 mL, 20:1) and cooled to 0 °C. $LiBH_4$ (81 mg, 3.70 mmol, 1.5 equiv.) was added subsequently to the solution and stirred for 1 h. After completion, the reaction was quenched with HCl (1M, 10 mL), and the aqueous layer was extracted with CH_2Cl_2 (3 × 20 mL). The combined organic layers were dried over $MgSO_4$, filtered, and concentrated under reduced pressure. The crude product was dissolved in CH_2Cl_2 and purified by flash chromatography on silica gel (CH_2Cl_2:Et_2O = 99:1). The reduced alcohol product 2*R*,3*R*-3-((tert-butyldimethylsilyl)oxy)-2-methylpentan-1-o**l** was obtained as a colorless oil (420 mg, 1.80 mmol, 73.5 %). **R_f** = 0.70 (CH_2Cl_2:Et_2O = 99:1); **^{1}H NMR** (400 MHz, $CDCl_3$) δ = 3.72-3.66 (m, 2H, OHC*H2*), 3.52 (dd, *J* = 10.6, 5.1 Hz, 1H, C*H*OTBS), 2.65 (bs, 1H, O*H*), 2.06-1.87 (m, 1H, OHCH2C*H*), 1.58-1.47 (m, 2H, CH3C*H2*COTBS), 0.91-0.88 (m, 12H, OSi(CH3)2C(C*H3*)*3*, *CH3*CH2), 0.81 (d, *J* = 7.0 Hz, 3H, C*H3*CH), 0.09 (s, 3H, OSi(C*H3*)2C(CH3)3), 0.07 (s, 3H, OSi(C*H3*)2C(CH3)3).

2*S*,3*R*-3-((tert-butyldimethylsilyl)oxy)-2-methylpentanal[5]

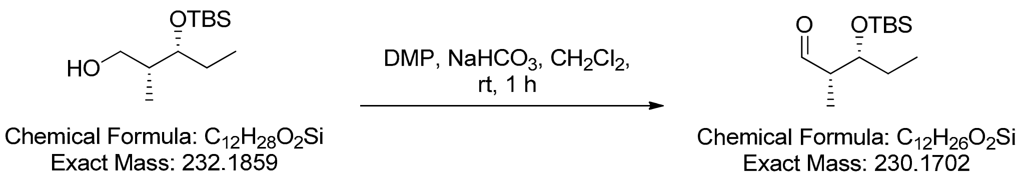

2*R*,3*R*-3-((tert-butyldimethylsilyl)oxy)-2-methylpentan-1-ol (400 mg, 1.72 mmol, 1.0 equiv.) was dissolved in CH_2Cl_2 (20 mL) and $NaHCO_3$ (84.0 mg, 1.03 mmol, 0.6 equiv.) was added. To the reaction mixture, Dess-Martin periodinane (873 mg, 2.06 mmol, 1.2 equiv.) was added subsequently, and stirring continued for 1 h at rt. The reaction was quenched by the addition of aq. $Na_2S_2O_3$ (10 mL) and stirred for 1 h. The layers were separated and the aqueous layer was extracted with CH_2Cl_2 (3x 15 mL). The combined organic layers were dried over $MgSO_4$, filtered, and concentrated under reduced pressure. The crude product was dissolved in CH_2Cl_2 and purified by flash chromatography (PE:EtOAc = 20:1) to furnish aldehyde 2*S*,3*R*-3-((tert-butyldimethylsilyl)oxy)-2-methylpentanal as a colorless liquid (305 mg, mmol, 74 %), which was used immediately in the next step. $\mathbf{R_f}$ = 0.81 (CH_2Cl_2:Et_2O = 99:1); **^{1}H NMR** (400 MHz, $CDCl_3$) δ = 9.77 (d, *J* = 0.8 Hz, 1H C*H*O), 4.03 (td, *J* = 6.5, 3.7 Hz, 1H, C*H*OTBS), 2.46 (qdd, 6.9, 3.6, 1.0 Hz, 1H, COC*H*CH_3), 1.55-1.47 (m, 2H, $CH_3C\mathit{H_2}$COTBS), 1.06 (d, *J* = 6.9 Hz, 3H, $C\mathit{H_3}$CH), 0.91-0.89 (m, 3H, $C\mathit{H_3}CH_2$), 0.86 (s, 9H, $OSi(CH_3)_2C(C\mathit{H_3})_3$), 0.06 (s, 3H, $OSi(C\mathit{H_3})_2C(CH_3)_3$), 0.04 (s, 3H, $OSi(C\mathit{H_3})_2$ $C(CH_3)_3$); **^{13}C NMR** (100 MHz, $CDCl_3$) δ = 205.6 (t, *C*HO), 73.5 (t, *C*HOTBS), 50.9 (t, CO*C*HCH_3), 27.5 (s, *C*H_2CH_3), 25.9 (p, $OSi(CH_3)_2C(\mathit{C}H_3)_3$), 18.1 (q, $OSi(CH_3)_2\mathit{C}(CH_3)_3$), 10.2 (p, *C*$H_3CH_2$), 7.7 (p, *C*$H_3$CH), -4.0 (p, $OSi(\mathit{C}H_3)_2C(CH_3)_3$), -4.5 (p, $OSi(\mathit{C}H_3)_2C(CH_3)_3$).

R-4-benzyl-3-((2*R*,3*S*,4*R*,5*R*)-5-((tert-butyldimethylsilyl)oxy)-3-hydroxy-2,4-dimethylheptanoyl)oxazolidin-2-one[6]

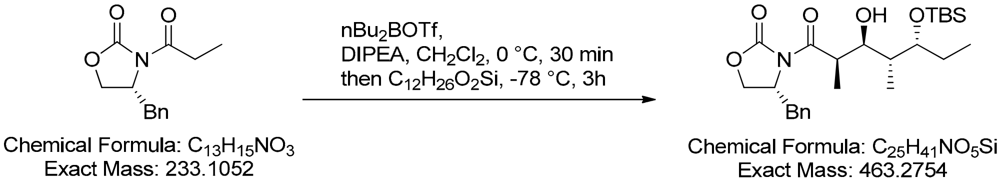

R-4-benzyl-3-propionyloxazolidin-2-one (424 mg, 1.82 mmol, 1.2 equiv.) was dissolved in anhydrous CH_2Cl_2 (10 mL) and cooled to 0 °C. To the stirred solution *n*Bu_2BOTf (1.0 M in CH_2Cl_2, 1.84 mL, 1.84 mmol, 1.2 equiv.) was added dropwise over 15 min. After addition, DIPEA (0.45 mL, 2.25 mmol, 1.4 equiv.) was added, and stirring continued for an additional 15 min. Then the solution was cooled to -78 °C and 2*S*,3*R*-3-((tert-butyldimethylsilyl)oxy)-2-methylpentanal (300 mg, 1.30 mmol, 1.0 equiv.) was added dropwise. After addition, the solution was stirred for 3h at -78 °C, before the reaction was allowed to warm to 0 °C. The reaction was quenched with potassium phosphate buffer (pH 7.1, 8 mL), MeOH (4 mL), and MeOH/30 %-H_2O_2 (12 mL, 2:1). After stirring 1 h at 0 °C the aqueous layer was extracted with CH_2Cl_2 (3 × 20 mL). The combined organic layers were washed with saturated $NaHCO_3$- and NaCl-Solution, dried over $MgSO_4$, filtered, and concentrated under reduced pressure. The crude product was dissolved in CH_2Cl_2 and purified by flash chromatography (PE:EtOAc = 20:1). The aldol product was obtained as a colorless oil (428 mg, 0.92 mmol, 71 %). **R_f** = 0.75 (PE:EE 4:1), **^{1}H NMR** (400 MHz, $CDCl_3$) δ = 7.36-7.26 (m, 3H, Bn), 7.23-7.20 (m, 2H, Bn), 4.72-4.66 (m, 1H, N*CH*Bn), 4.24-4.15 (m, 2H,O*CH2*CHBn), 4.02 (d, *J* = 9.8 Hz, 1H, C*H*OH), 3.89-3.83 (m, 1H, C*H*OTBS), 3.35 (dd, *J* = 13.3, 3.2 Hz, BnC*H2*), 2.75 (dd, *J* = 13.3, 9.8, Hz, 1H BnC*H2*), 1.83 (m, 1H, COC*H*COH), 1.59-1.51 (m, 2H, CH_3C*H2*CH) 1.22 (d, *J* = 6.9 Hz, C*H3*CHCOH) 0.90 (s, 9H, OSi(CH*3*)2C(C*H3*)*3*), 0.85 (d, *J* = 7.1 Hz, 3H, C*H3*CHCOTBS) 0.12 (s, 3H, OSi(C*H3*)2C(CH_3)3), 0.08 (s, 3H, OSi(C*H3*)2C(CH_3)3).

3*R*,4*S*,5*R*,6*R*-6-ethyl-4-hydroxy-3,5-dimethyltetrahydro-2H-pyran-2-one (Triketide lactone)[7,8]

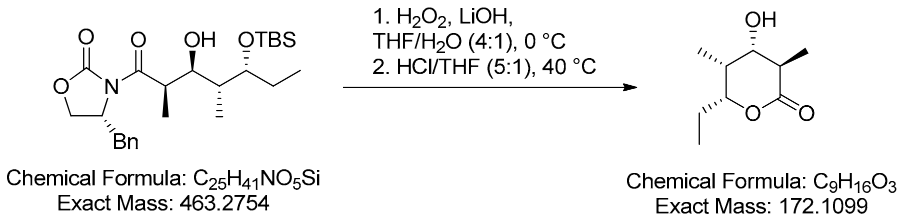

R-4-benzyl-3-((2*R*,3*S*,4*R*,5*R*)-5-((tert-butyldimethylsilyl)oxy)-3-hydroxy-2,4-dimethylheptan-oyl)oxazolidin-2-one (200 mg, 0.43 mmol, 1.0 equiv.) was dissolved in THF:H_2O (3 mL, 4:1) and cooled to 0 °C. To the solution, H_2O_2 (30 % in H_2O, 170 μL, 1.72 mmol, 4.0 equiv.) was first added dropwise followed by LiOH (1M in H_2O, 690 μL, 0.69 mmol, 1.6 equiv.). After stirring for 2h the volatiles were removed under reduced pressure, the remaining aqueous layer was adjusted to pH 1 with 1M HCl and extracted with CH_2Cl_2 (3 × 3 mL). The organic layers were combined and concentrated under reduced pressure. The residue was then dissolved in 1M HCl:THF (5 mL, 5:1) and heated to 40 °C for 5 h. The pH of the aqueous phase was adjusted to 7 with 1M NaOH and extracted with CH_2Cl_2 (3 × 5 mL). The combined organic layers were dried over $MgSO_4$, filtered, and concentrated under reduced pressure. The crude product was dissolved in CH_2Cl_2 and purified by flash column chromatography. The triketide lactone was obtained as a colorless oil (52 mg, 0.30 mmol, 67 % yield). $\mathbf{R_f}$ = 0.32 (PE:EtOAc 2:1); **^{1}H NMR** (400 MHz, $CDCl_3$) δ = 4.13 (ddd, J = 8.3, 6.3, 2.3 Hz, 1H, C*H*O), 3.82 (dt, J = 10.2, 4.5 Hz, 1H, C*H*OH), 2.46 (qd, J = 10.3 , 7.1 Hz, 1H, $CH_3CHC(O)$), 2.16 (qdd, J = 7.0, 4.1, 2.4 Hz, 1H, CH_3CHCO) 1.87-1.76 (m, 2H, C*H*CH_3, O*H*), 1.62-1.50 (m, 2H, CH_2CH_3), 1.40 (d, J = 7.1 Hz, 3H, $CH_3CHC(O)$), 1.00 (t, J = 7.5 Hz, 3H, CH_3CH), 0.96 (d, J = 7.1 Hz, 3H, CH_3CH_2); ***m/z* (ES+)**, 345.4 $[M_2]H^+$, 195.2 [M]Na+, 173.2 $[M]H^+$, 155.2 $[M-H_2O]H^+$, 137.2 $[M-2H_2O]H^+$.

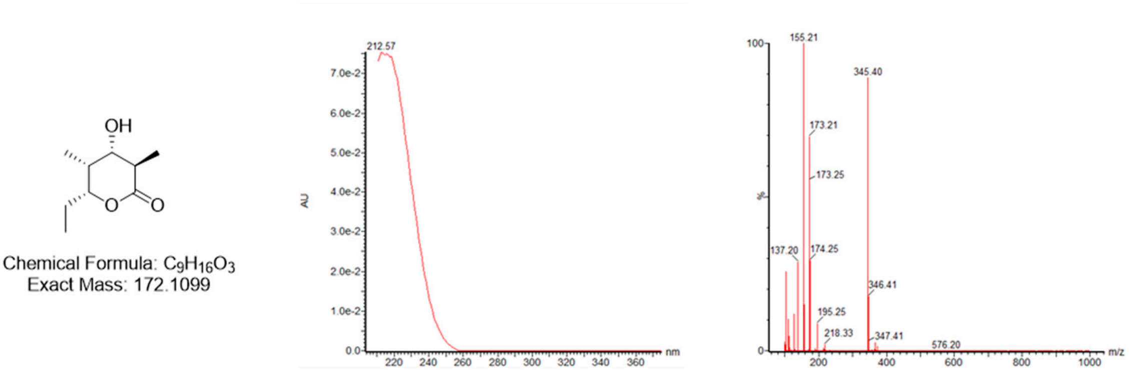

Fig. S7.1 Structure (left), UV Spectrum (middle), and fragmentation pattern (ES+ right) of triketide lactone TKL.

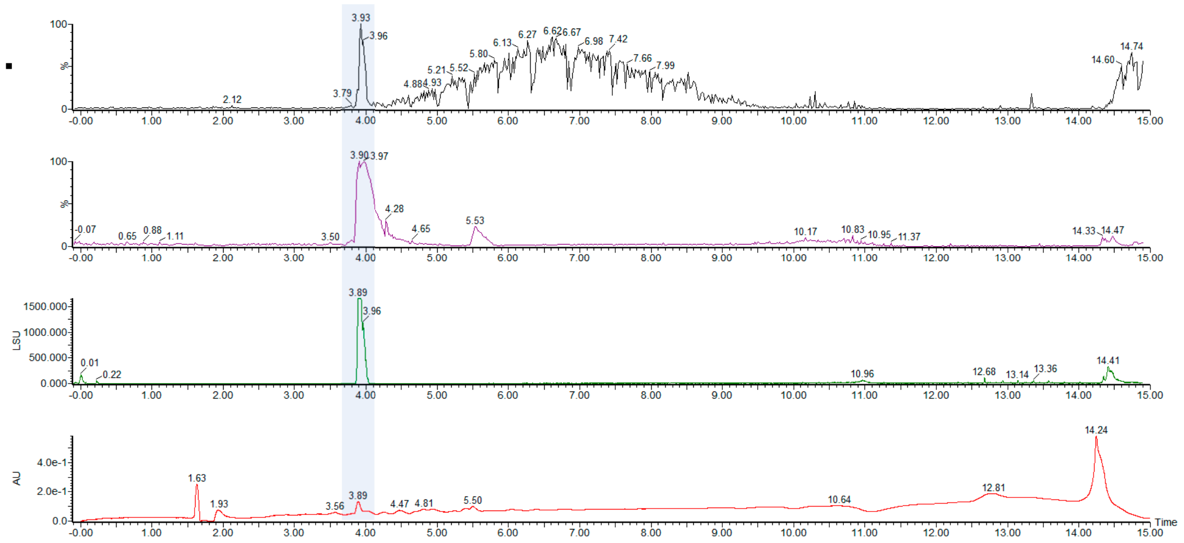

Fig. S7.2 LC-MS analysis of purified triketide lactone TKL, UV (diode array scan, 200-600 nm, red), ELSD (electron light scattering, green), ES+ (violet), ES- (black).

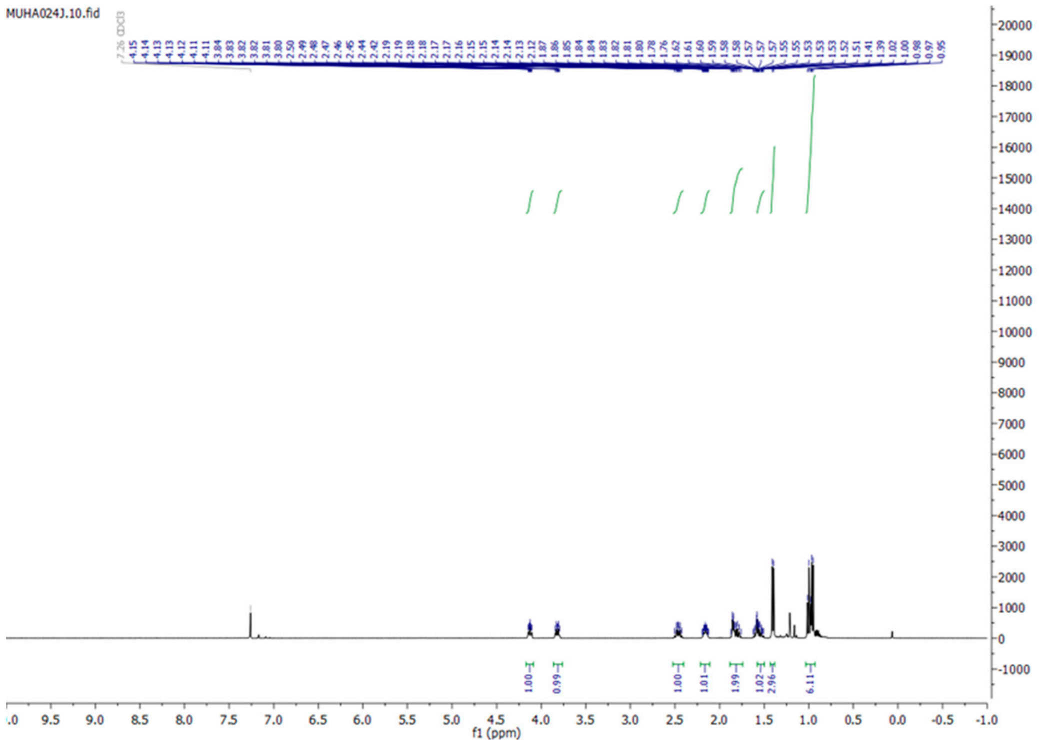

Fig. S7.3 ^{1}H NMR of triketide lactone TKL recorded at 400 MHz in $CDCl_3$.

8. Determination and Quantification of TKL

The authentic triketide lactone TKL was chemically synthesized. Using it as the control, the crude extract from *A. oryzae* was comparatively analyzed with the method of selected ion recording (SIR) by LCMS (Figure S8.1). Only the peak with $[M+H]^+$ 173 was set to record on ES+ TIC chromatogram. It turned out that peaks recorded on both of SIR chromatograms had the identical retention time (Figure S8.1 A-B). Subsequently, the authentic TKL with the concentration of 20 $\mu g \cdot mL^{-1}$ was mixed with the crude extract from *A. oryzae* at the volume ratio of 1:1, followed by SIR analysis on LCMS. It also demonstrated that it was a homogenous peak, not splitting into two peaks (Figure S8.1 C). Moreover, the fragmentation pattern of the authentic TKL was also consistent with that of the newly-formed product from *A. oryzae*. The parent ion *m/z* 173 and the daughter ion *m/z* 155 were predominantly present on both of samples (Figure S8.1 D-E).

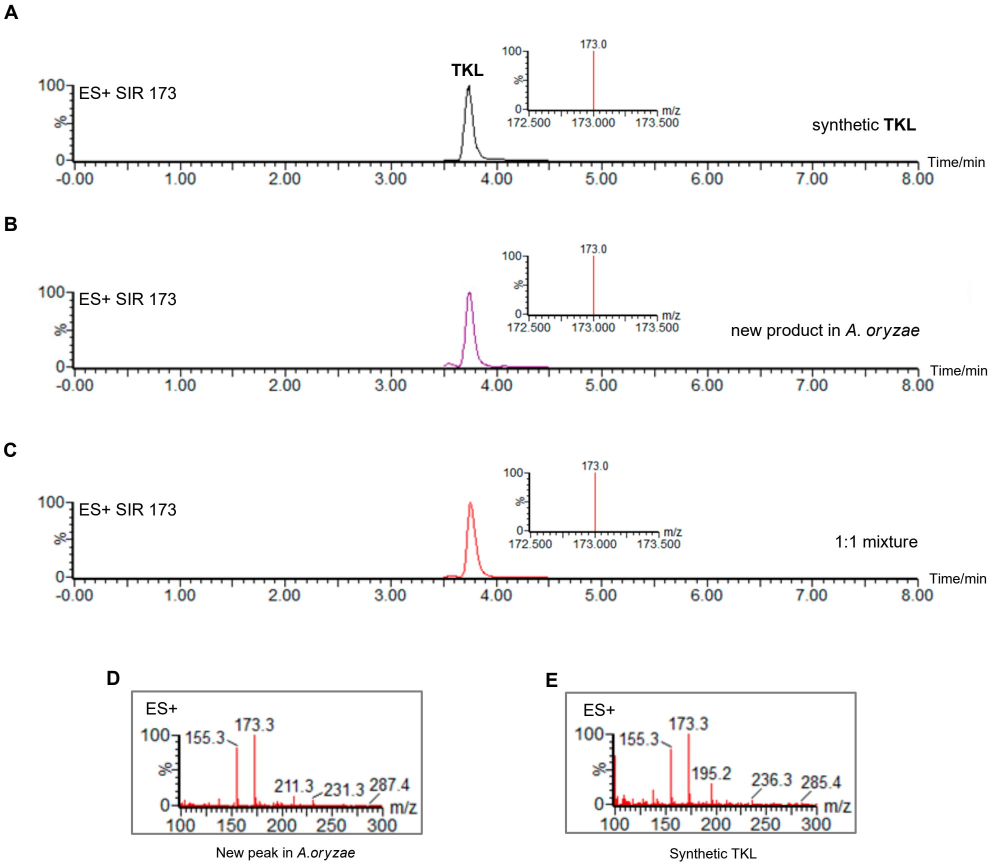

Figure S8.1 Comparative analysis of the synthetic **TKL** and the crude extract from *A. oryzae* transformant: **A**, ES+ SIR chromatogram of synthetic **TKL**; **B**, ES+ SIR chromatogram of crude extract from *A. oryzae* transformant; **C**, ES+ SIR chromatogram of 1:1 mixture of samples above; D, ES+ TIC fragmentation of the newly-formed product in *A. oryzae*; E, ES+ TIC fragmentation of the synthetic TKL.

A standard method was established for the titer measurement of triketide lactone using the authentic compound. First, the authentic compound was dissolved in methanol and diluted to different concentrations from 2.5 μg·mL^{-1} to 200 μg·mL^{-1}. Each concentration was analyzed and the corresponding product peak on SIR (single ion recording) LCMS chromatogram was integrated. Then, a calibration curve was made based on the concentrations and peak areas of the authentic compound (Figure S8.2). Then, a standard extraction workflow was determined. The transformant was inoculated in 100 mL DPY media. After 4 days of cultivation, 50 mM sodium propionate was added into the culture. The culture with propionate was incubated overnight. Next, the five-day culture was extracted with the same volume of ethyl acetate twice. The organic phase was gathered and evaporated to dryness. The concentrated extract was re-dissolved in 1.5 mL methanol. Subsequently, the extract suspension was diluted 10 times with methanol. The sample was centrifuged at 14,000 × g for 5 min. Lastly, the supernatant was collected and analyzed by LCMS using the SIR detection method.

The extract from 100 mL of the transformant culture were applied to the quantitative analysis based on the calibration curve. By calculation, the titer of the triketide lactone product in *A. oryzae* was approximately 0.6 mg·L^{-1}.

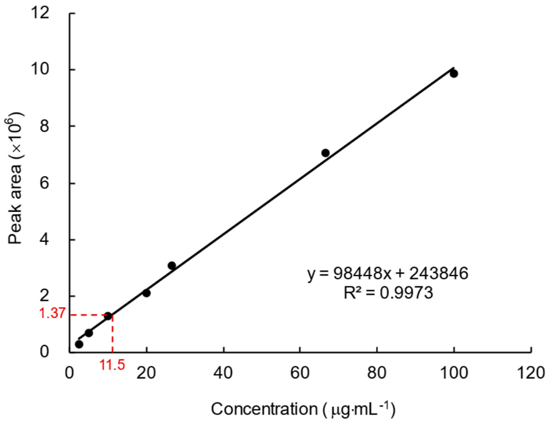

Figure S8.2 The establishment of the calibration curve for the titer calculation of the product. The red numbers are the calculated values of the product produced by *A. oryzae* transformant.

9. Deletion of Propionyl-CoA Degradation Pathways

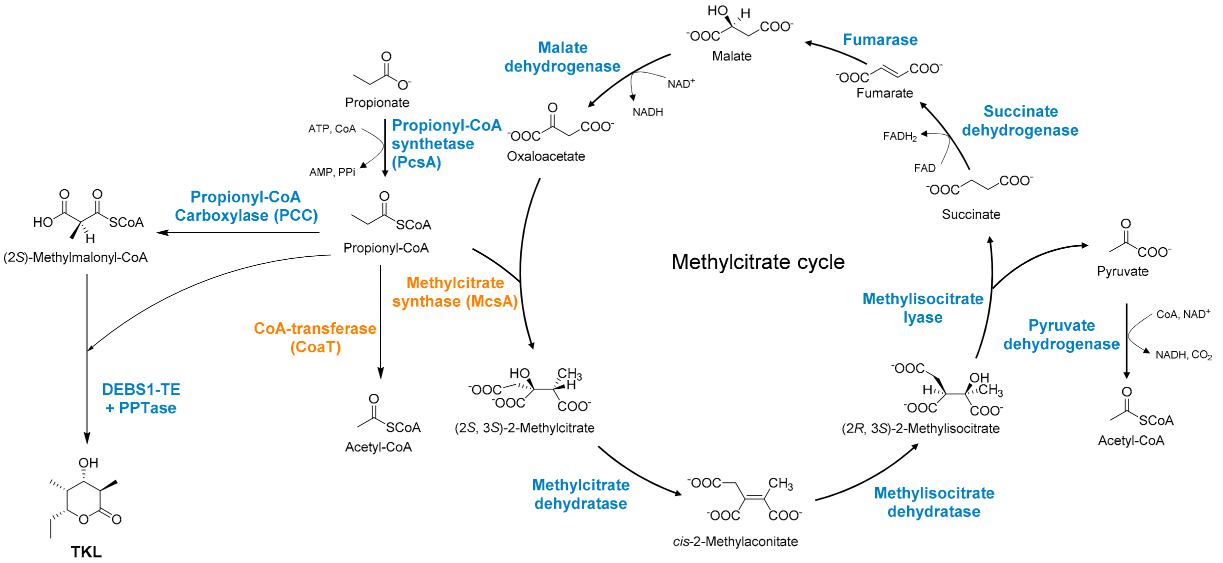

Scheme S9.1 Overall metabolic pathways of DEBS1-TE in engineered *A. oryzae* transformant. The orange enzymes represent the degradation pathway of the starter unit propionyl-CoA.

To block propionyl-CoA degradation pathways in *A. oryzae* NSAR1, gene knockout were considered with auxotrophic markers. Given that *A. oryzae* NSAR1 is a quadruple auxotrophic strain (*niaD*⁻, *argB*⁻, *adeA*⁻, and *sC*⁻), the selection marker *argB* was first used for *mcsA* gene knockout.

The *mcsA* gene knockout cassette with the auxotrophic marker *argB* was constructed by yeast recombination, and the bipartite gene knockout-based fungal transformation was carried out as previously published protocols (Figure S9.2 A). [9,10] After transformant screening, 48 transformants were obtained followed by genomic DNA extraction. Next, all genomic DNA samples were identified by PCR using multiple primers. 3 out of 48 transformants were determined as positive *mcsA* mutants (JFI98D, JFI98E and JFI98L). When using primers JF74F and JF74R, an expected PCR band was observed with the size of 3304 bp, that is distinctively different from 1758 bp in the NSAR1 strain (Figure S9.2 B). When one of primers was located within *argB*, the accurate PCR bands were also obtained in three positive transformants (Figure S9.2 C-D). Evidently, it demonstrated that *mcsA* gene had been deleted in this mutant strain.

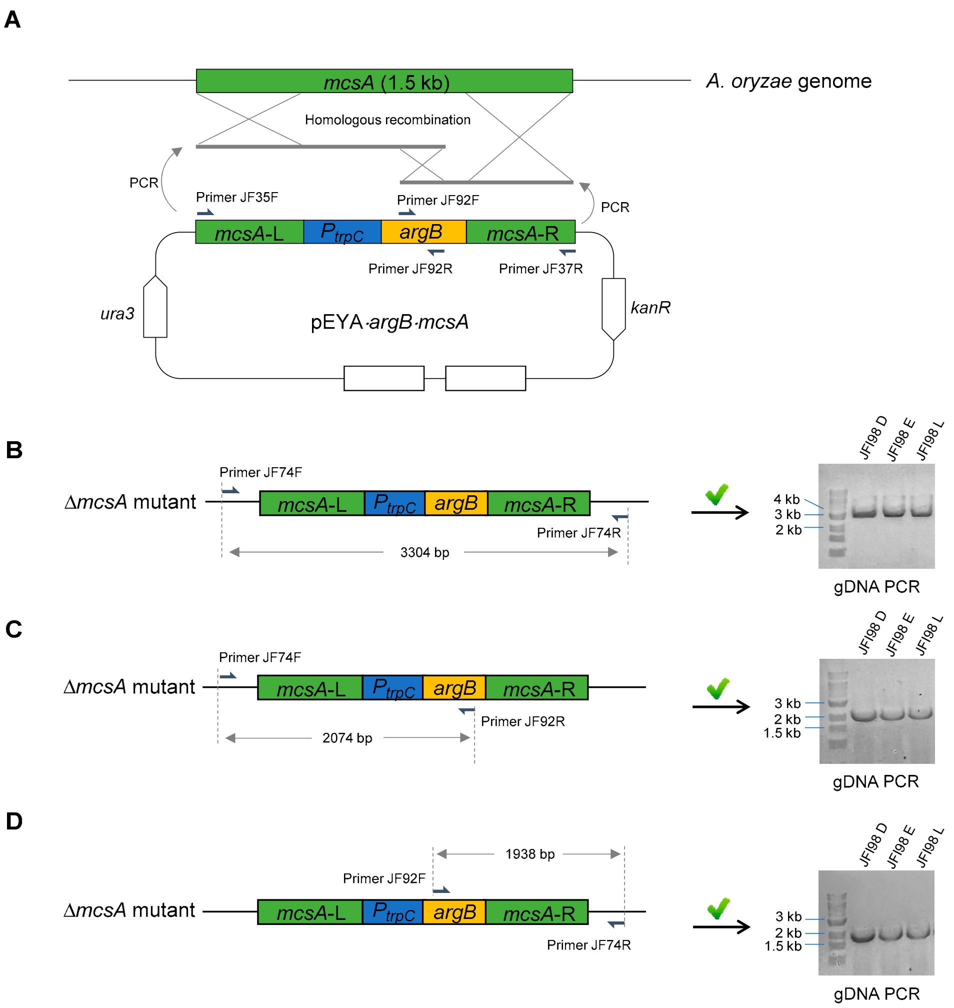

Figure S9.2 Gene knockout of the *mcsA* target using the auxotrophic selection marker *argB*: **A**, the construction of the *mcsA* gene knockout cassette and the homologous recombination with *A. oryzae* genome; **B**, the identification of the whole gene cassette in the *mcsA* transformants by gDNA PCR using primers JF74F/JF74R; **C**, the identification of the left segment of the *mcsA* gene cassette using primers JF74F/JF92R; **D**, the identification of the right segment of the *mcsA* gene cassette using primers JF92F/JF74R.

The single *coaT* gene was attempted to delete as shown in Figure S9. Two overlapped PCR fragments were amplified from the *coaT* gene knockout cassette and then introduced into *A. oryzae* NSAR1 based on a bipartite gene knockout strategy (Figure S9.3 A).[3] After fungal transformation, 31 transformants were obtained and screened by genomic DNA PCR. Lastly, only one *coaT* mutant transformant JFI108B was found. The gene identification showed distinct differences from other transformants by PCR. Using primers JF105F and JF105R, the native *coaT* gene at 2890 bp was absent compared with other transformants (Figure S9.3 B). The identification of the first half of *coaT* gene cassette was ambiguous while the second half was determined in the transformant

JFI108B (Figure S9.3 C-D). Therefore, it is speculated that the integrity of *coaT* gene was disrupted *in vivo*.

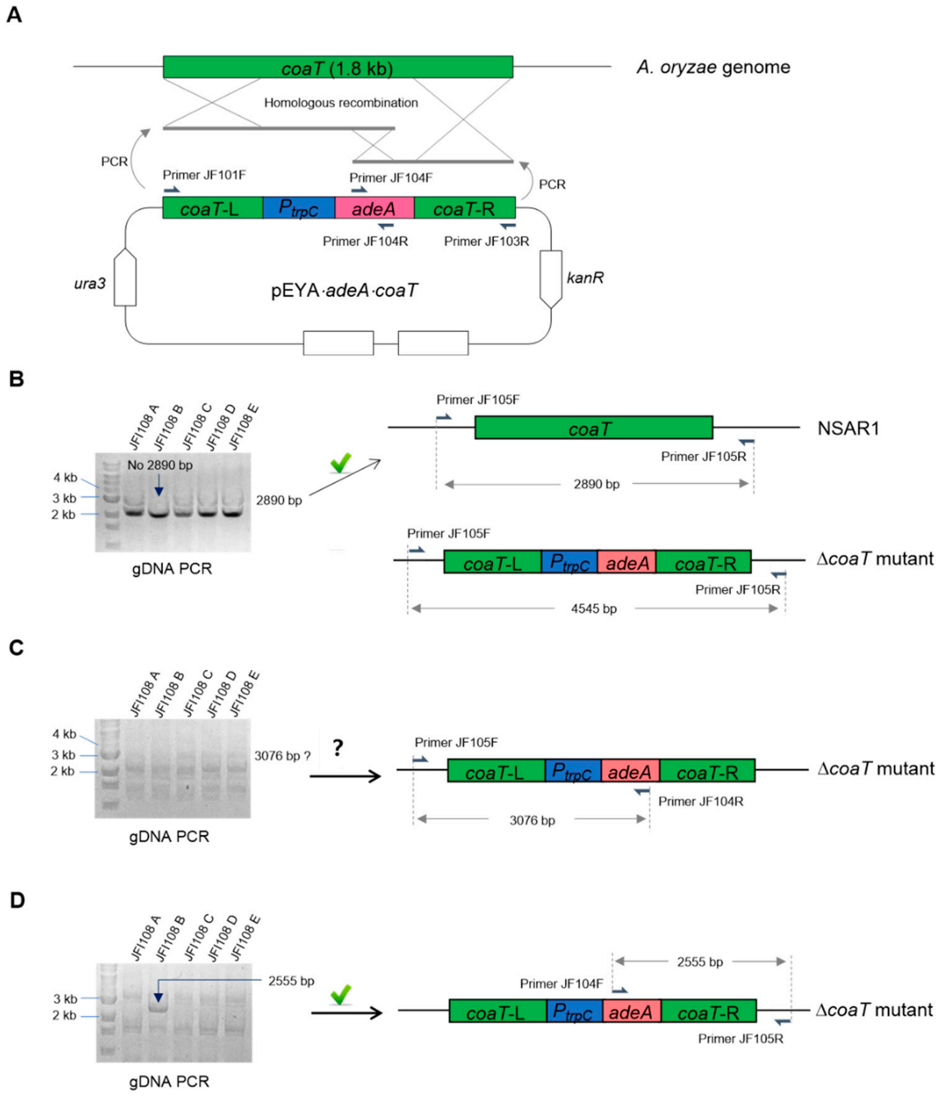

Figure S9.3 Gene knockout of the *coaT* target using the auxotrophic selection marker *adeA*: **A**, the construction of the *coaT* gene knockout cassette and the homologous recombination with *A. oryzae* genome; **B**, the identification of the whole gene cassette in the *coaT* transformants using primers JF105F/JF105R; **C**, the identification of the left segment of the *coaT* gene cassette using primers JF105F/JF104R; **D**, the identification of the right segment of the *coaT* gene cassette using primers JF104F/JF105R.

10. Media, Solutions, Strains, Plasmids and Primers

Table S3 Media used in the study.

Media	Composition (%(*v/w*))	Ingredients
GN	2.00	D(+)-Glucose Monohydrate
	1.00	Nutrient broth Nr. 2 from Oxoid
LB	0.50	Yeast extract
	1.00	Tryptone
	0.50	Sodium chloride
SM-URA	0.17	Yeast nitrogen base
	0.50	Ammonium sulfate
	2.00	D(+)-Glucose Monohydrate
	0.077	Complete supplement mixture minus Uracil
CZD/S	3.50	Czapek Dox broth
	18.22	D-Sorbitol
	0.10	Ammonium sulfate
	0.05	Adenine
	0.15	L-Methionine
CZD/S without methionine	3.50	Czapek Dox broth
	18.22	D-Sorbitol
	0.10	Ammonium sulfate
	0.05	Adenine
DPY	2.00	Dextrin from potato starch
	1.00	Polypeptone
	0.50	Yeast extract
	0.50	Monopotassium phosphate
	0.50	Magnesium sulfate hexahydrate

Table S4 Solutions used in the study.

Solutions	Concentration	Ingredients
Carbenicillin	50 $mg{\cdot}mL^{-1}$	Carbenicillin
Kanamycin	50 $mg{\cdot}mL^{-1}$	Kanamycin
PEG solution	50 % (*w/v*)	Polyethylene glycol 3350
LiAc	1 M	Lithium acetate
Salmon testis DNA solution	2 $mg{\cdot}mL^{-1}$	Salmon testis DNA
Lysing solution for *A. oryzae*	10 $mg{\cdot}mL^{-1}$	*Trichoderma* lysing enzyme
	0.8 M	NaCl
Solution I (pH 7.5)	0.8 M	NaCl
	10 mM	$CaCl_2$
	50 mM	Tris-HCl, 7.5
Solution II (pH 7.5)	60 % (*w/v*)	Polyethylene glycol 3350
	0.8 M	NaCl
	10 mM	$CaCl_2$
	50 mM	Tris-HCl, 7.5

Table S5 Strains used in the study.

**Strains**	**Genotype**	**Origin**	**Function**
E. coli One Shot^{TM} Top10	*F*$^-$ *mcrA Δ(mrr-hsdRMS-mcrBC) Φ80lacZΔM15 Δ lacX74 recA1 araD139 Δ(ara-leu)7697 galU galK rpsL (Str*^{R}*) endA1 nupG*	Thermo Fisher Scientific	Plasmid construction
E. coli One Shot^{TM} *ccdB* Survival^{TM} 2 T1^R	*F*$^-$*mcrA Δ(mrr-hsdRMS-mcrBC) Φ80lacZΔM15 ΔlacX74 recA1 araΔ139 Δ(ara-leu)7697 galU galK rpsL (Str*^{R}*) endA1 nupG fhuA::IS2*	Thermo Fisher Scientific	Plasmid construction
E. coli One Shot™ OmniMAX™ 2 T1^R	*F´ {proAB lacIq lacZΔM15 Tn10(TetR) Δ(ccdAB)} mcrA Δ(mrr hsdRMS-mcrBC) Φ 80(lacZ)ΔM15 Δ(lacZYA-argF)U169 endA1 recA1 supE44 thi-1 gyrA96 relA1 tonA panD*	Thermo Fisher Scientific	Plasmid construction
S. cerevisiae CEN.PK	*MATa/α ura3-52/ura3-52 trp1-289/trp1-289 leu2-3_112/leu2-3_112his3 Δ1/his3 Δ1 MAL2-8C/MAL2-8C SUC2/SUC2*	Hahn group, Hannover	Plasmid construction
A. oryzae NSAR1	*argB*$^-$ *sC*$^-$ *adeA*$^-$ *niaD*$^-$	Lazarus group, Bristol	Metablite production

Table S6 Plasmids used and constructed in this study.

**No.**	**Name (inserted genes)**	**Marker genes**	**Expression host**
**I**	pTYGS·*arg*	*ampR*, *ura3*, *argB*	-
**II**	pTYGS·*arg*·*pccABE*	*ampR*, *ura3*, *argB*	-
**III**	pUC57·*debs1te*·*f1*	*ampR*	-
**IV**	pUC57·*debs1te*·*f2*	*ampR*	-
**V**	pUC57·*debs1te*·*f3*	*ampR*	-
**VI**	pUC57·*debs1te*·*f4*	*ampR*	-
**VII**	pEYA	*kanR*, *ura3*	-
**VIII**	pEYA·*debs1te*	*kanR*, *ura3*	-
**IX**	pTYGS·*arg*·*debs1te*-*pccABE*	*ampR*, *ura3*, *argB*	*A. oryzae* NSAR1
**X**	pEYA·*debs1te*·*egfp*	*ampR*, *ura3*, *argB*	*A. oryzae* NSAR1
**XI**	pTYGS·*arg*·*debs1te*·*egfp*	*ampR*, *ura3*, *argB*	*A. oryzae* NSAR1
**XII**	pTYGS·*arg*·*pccA*·*egfp*	*ampR*, *ura3*, *argB*	*A. oryzae* NSAR1
**XIII**	pTYGS·*arg*·*pccB*·*egfp*	*ampR*, *ura3*, *argB*	*A. oryzae* NSAR1
**XIV**	pTYGS·*arg*·*pccE*·*egfp*	*ampR*, *ura3*, *sC*	-
**XV**	pTYGS·*met*	*ampR*, *ura3*, *sC*	*A. oryzae* NSAR1
**XVI**	pTYGS·*met*·*sfp*	*ampR*, *ura3*, *sC*	*A. oryzae* NSAR1
**XVII**	pTYGS·*met*·*sepptII*	*ampR*, *ura3*, *argB*	*A. oryzae* NSAR1
**XVIII**	pTYGS·*arg*·*debs1te*·*egfp*·*pccABE*	*ampR*, *ura3*, *sC*	*A. oryzae* NSAR1
**XIX**	pTYGS·*met*·*sepptII*-*pcsA*	*kanR*, *ura3*	-

No.	Name (inserted genes)	Marker genes	Expression host
**XX**	pEYA·*argB*·*mcsA*	*kanR*, *ura3*	-
**XXI**	pEYA·*adeA*·*coaT*	*kanR*, *ura3*	-

Table S7 Primers used in this study.

No.	Sequence (5′-3′)	Information
JF4F	CTGCGCTTCTGATATCGGGT	*pcsA* test primer
JF4R	GTCCCTGTATCATGCCTCCG	*pcsA* test primer
JF25F	CCCGATTGTTTAATGATGACGAG	*pcsA* test primer
JF20R	CCAATCGGTGACAGGCTCGAA	*debs1te* test primer
JF22F	CGATCGCGGCCATCCTCCTCG	*debs1te* test primer
JF19F	ATACCCCTGCCACCTCCTACC	*pccA* test primer
JF23R	CTCCTCGACGAGCTTCTGATGG	*pccA* test primer
JF24R	ACGACGAGGGAGATCTGAGG	*pccB* test primer
JF6F1	CCATGCCCATGCGAACCACTC	*mcsA* test primer
JF6R	GGTAGAAGAGAACACCAGACGC	*mcsA* test primer
JF26R	GGTGGGACTTCTCGGCCAATT	*mcsA* test primer
JF18F	GGCGGCCTTCCTACCTCAAG	*coaT* test primer
JF18R	GAGGAGACAGACCACGCACG	*coaT* test primer
JF27R	GCACCAGGGATGATACCACCAT	*coaT* test primer
JF35F	ATGGCCATGCCCATGCGAAC	*mcsA* overlap L primer
JF37R	TCATTTCTTCAGGCCCAGGA	*mcsA* overlap R primer
JF74F	CGACACAAAGCAGTGCTCCTGTGTTG	*mcsA* mutant test primer
JF74R	CTGGCGTCTATGCAAATGGGCATATGGC	*mcsA* mutant test primer
JF92F	ACTCCTCGCAAACCATGCC	*argB* primer
JF92R	TGGTTTGCAGAAGCTTTCCTG	*argB* primer
JF101F	AAGATAGACGAAAAGAAAAC	*coaT* overlap L primer
JF103R	GAGGTTGTTCTTGTTGGGAG	*coaT* overlap R primer
JF104F	GGTTGACTGCCTCAGGCCTGC	*adeA* primer
JF104R	CGACCGATCTCGTACGAGTCC	*adeA* primer
JF105F	GAGAGCCGAGAATACGCCGAG	*coaT* mutant test primer
JF105R	GAGATGTGGGTGCTCATGCTGG	*coaT* mutant test primer
146	GTAAAACGACGGCCAG	General primer (pE·YA)
147	TGCTTGGAGGATAGCAACCG	General primer (P_{amyB})
148	GGGGATGACAGCAGTAACGA	General primer (T_{amyB})
168	CTTCCGTCCTCCAAGTTAGT	General primer (P_{eno})
189	CTTTGTGCAGCTCAGAGGTT	General primer (T_{eno})
169	ACCATCTTTCGATAATGTGT	General primer (T_{eno})
170	ACTTCATCGCAGCTTGACTA	General primer (P_{gpdA})
171	TCTTTCATTATCTTGCGAAC	General primer (T_{gpdA})

11. References

1. Gietz, R. D.; Schiestl, R. H., *Nat. Protoc*. **2007**, 2, 35–37.
2. Pahirulzaman, K. A. K.; Williams, K.; Lazarus, C. M., A Toolkit for Heterologous Expression of Metabolic Pathways in *Aspergillus oryzae*, 1st ed.; Elsevier Inc., 2012; Vol. 517.
3. D. E. Cane, R. H. Lambalot, P. C. Prabhakaran, W. R. Ott, *J. Am. Chem. Soc.,* **1993**, *115*, 522–526.
4. C.-L. Kao, S. A. Borisova, H. J. Kim, H. Liu, *J. Am. Chem. Soc.,* **2006**, *128*, 5606–5607.
5. N. Schläger, A. Kirschning, *Org. Biomol. Chem.,* **2012**, *10*, 7721–7729.
6. D. A. Evans, A. S. Kim, R. Metternich, V. J. Novack, *J. Am. Chem. Soc.,* **1998**, *120*, 5921–5942.
7. B. J. Beck, C. C. Aldrich, R. A. Fecik, K. A. Reynolds, D. H. Sherman, *J. Am. Chem. Soc.,* **2003**, *125*, 12551–12557.
8. M. J. B. Brown, J. Cortes, A. L. Cutter, P. F. Leadlay, J. Staunton, *J. Chem. Soc. Chem. Commun.,* **1995**, *0*, 1517–1518.
9. C. Wang, K. Becker, S. Pfütze, E. Kuhnert, M. Stadler, R. J. Cox, E. Skellam, *Org. Lett.,* **2019**, *21*, 8756–8760.
10. T. T. Dao, K. M. J. de Mattos-Shipley, I. M. Prosser, K. Williams, M K. Zacharova, C. M. Lazarus, C. L. Willis, A. M. Bailey, *Front. Fungal Biol*., 2021, **2**: 632542.
